# AB4-Loaded Nanomicelle Hydrogel Promotes Targeting of the Dysregulated Diabetic Wound Microenvironment via Coordinated Multistage Repair

**DOI:** 10.3390/gels12080722

**Published:** 2026-08-14

**Authors:** Xue Shao, De-Jing Ma, Ya-Ni Zhang, Bang-Yun Liu, Yi-Fei Gao, Ge Zhang, Zi-Yan Hua, Yan-Yun Yang, Xue-Tao Li, Liang Xu

**Affiliations:** School of Pharmacy, Liaoning University of Traditional Chinese Medicine, Dalian 116600, China

**Keywords:** *Panax notoginseng* oligosaccharide (PNO), anti-inflammatory, combined therapy, microenvironment modulation, drug-carrier integration

## Abstract

(1) Background: Impaired diabetic wound healing stems from systemic dysregulation of the wound-healing cascade under hyperglycemic conditions, producing a disordered microenvironment marked by sustained inflammation, defective angiogenesis, and aberrant extracellular matrix remodeling, multifactorial, multistage pathological interactions demanding multi-target intervention. (2) Methods: We constructed a multifunctional nanocomposite hydrogel dressing (PGAs@CDV) based on a “drug-carrier integration” strategy, targeting the dysregulated hemostasis, inflammation, and proliferation phases of diabetic wound healing. An amphiphilic micelle carrier (PNO-GA) was synthesized by covalently conjugating *Panax notoginseng* oligosaccharide with gallic acid, loaded with Anemoside B4 to yield drug-loaded nanomicelles (PGAs), embedded into a carboxymethyl chitosan-dopamine-vanillin hydrogel (CDV) matrix to form PGAs@CDV. We then examined how PGAs@CDV affected diabetic wound healing. (3) Results: In vitro, PGAs@CDV enhanced cell migration and angiogenic capacity, exhibited potent antioxidant activity, and promoted M1-to-M2 macrophage polarization. We tested PGAs@CDV in a streptozotocin-induced diabetic mouse wound model. Wounds treated with PGAs@CDV closed faster than those treated with the control, CDV, PNO@CDV, and AB4@CDV. Four readouts tracked this difference: hemostasis was quicker, inflammation was lower, more blood vessels formed, and collagen deposition was higher. At the pathway level, PGAs@CDV suppressed NF-κB signaling and activated PI3K/AKT/HIF-1α. These two arms map onto the anti-inflammatory and pro-angiogenic effects observed above. (4) Conclusions: This nanocomposite hydrogel integrates a bioactive carrier with a therapeutic payload to enable coordinated intervention across multiple phases of diabetic wound repair. By combining structural support with sustained pharmacological activity, it offers a promising strategy for the treatment of chronic diabetic wounds.

## 1. Introduction

Diabetes affected an estimated 529 million people worldwide in 2021, corresponding to an age-standardized prevalence of 6.1%, and the global burden is projected to exceed 1.31 billion cases by 2050 [1]. Chronic diabetic wounds remain one of the most challenging complications of the disease, frequently resulting in infection, amputation, and substantial physical and economic costs [2,3,4]. Existing treatment options are far from satisfactory. Conventional debridement and drainage procedures are invasive and often increase patient discomfort; advanced therapies such as growth factors and stem-cell transplantation, although effective in selected settings, are expensive and technically demanding [5,6,7,8,9,10,11,12,13,14]. Thus, it is necessary to develop topical treatments that are efficacious, affordable, and able to broadly modulate the diabetic-wound area.

Hemostasis, inflammation, proliferation, and remodeling are the four stages of tissue recovery [2,12]. In diabetes, this process is chronically disrupted and prone to recurrence [6,12]. Systemic factors, including hyperglycemia, persistent inflammation, and neuropathy, combine with local pathological features, including barrier disruption, heavy exudation, ROS, and MMP overexpression, to collectively stall healing [15,16,17,18,19,20]. Intervention therefore needs to work on systemic and local factors, and at every stage of wound healing. This requires agents that act on multiple targets and stay active across multiple stages. Therefore, naturally derived compounds with broad bioactivity profiles (particularly Anemoside B4, AB4) have drawn attention.

AB4 is a triterpene saponin from *Pulsatilla chinensis*, with existing anti-inflammatory, antioxidant, and/or immunomodulatory functions; however, its clinical utility is constrained by its low water-solubility and limited bioavailability [21,22,23,24]. Polymeric micelles, constructed by the self-assembly of amphiphilic polymers into hydrophobic cores and hydrophilic shells, enable encapsulation and solubilization of such hydrophobic drugs. To build a micellar system combining delivery function with intrinsic bioactivity, we developed PNO-GA by covalently conjugating PNO with hydrophobic GA. The resulting amphiphile self-assembles into micelles with a GA hydrophobic core and a PNO hydrophilic shell; encapsulating AB4 within this core yields the drug-loaded nanomicelles PGAs, simultaneously improving AB4 solubility, hydrophilicity, and bioavailability.

We refer to this design as a drug-carrier integrated approach. Here, a bioactive carrier (PNO) is covalently linked to a bioactive small molecule (GA) to form an amphiphilic polymer (PNO-GA) that self-assembles into micelles, into whose hydrophobic core a third therapeutic agent (AB4) is loaded. Unlike conventional nanocarriers such as PEG or PLGA micelles, which are typically biologically inert, both the PNO-GA carrier and the AB4 cargo in this system are bioactive. The rationale for this design is threefold: to improve AB4 solubility through micelle formation, to prolong wound retention through the CDV hydrogel, and to achieve multistage repair through the combination of a bioactive carrier and cargo. The present study, however, does not directly compare PGAs@CDV with a simple physical mixture of PNO and AB4 lacking covalent conjugation, and we therefore make no claim that covalent linkage itself confers mechanistic or combinatorial superiority. Instead, we present PGAs@CDV as a multicomponent nanocomposite hydrogel and evaluate its efficacy empirically against formulations lacking one or more of its components.

PNO is a polysaccharide recovered from the residual liquor left after total *Panax notoginseng* saponins are extracted, which makes it a byproduct-derived material. It is built mainly of glucose and fructose, average 668 Da in molecular weight, and reaches 77.1% total sugar content. PNO does more than carry drug: it also strengthens the skin barrier and drives cell migration. Gallic acid (GA) is a plant polyphenol found in fruits and medicinal herbs, with a long-documented profile of antioxidant, anti-inflammatory, and antitumor activities [25,26,27]. Together in this system, PNO shapes the delivery matrix while GA supplies both structural and therapeutic functions, exemplifying the integrated drug-carrier design central to this study.

PGAs nanomicelles make AB4 water-soluble, but they clear from the wound quickly, so local drug levels drop and the therapeutic effect is short-lived. A hydrogel matrix addresses this. Once loaded into the hydrogel network, the PGAs release the drug slowly and the wound bed stays moist. The gel is biocompatibile and adheres to tissue. In diabetic wound models these features together have supported cell migration and tissue regeneration [28,29,30,31,32,33,34]. For comparison, Table A1 places the PGAs@CDV hydrogel alongside representative polysaccharide-based and synthetic wound dressings [31,32,33,34]. This comparison shows that PGAs@CDV combines rapid gelation, moderate swelling, and favorable mechanical properties with reliable therapeutic efficacy in a clinically relevant diabetic healing model, supporting its potential for diabetic wound applications. Notably, PNO serves a dual role as both carrier and active ingredient, and together with AB4 contributes anti-inflammatory and reparative effects.

This study constructed the multifunctional nanocomposite hydrogel PGAs@CDV for diabetic wound therapy. PNO-GA amphiphilic micelles were loaded with AB4 to form PGAs nanomicelles with improved solubility and delivery efficiency, then incorporated into a three-dimensional CDV hydrogel matrix to overcome rapid clearance and short wound-site retention. The resulting PGAs@CDV system exhibits wonderful biocompatibility and tissue adhesion, optimizes the diabetic condition, attenuates inflammation, improves angiogenesis and collagen deposition, and accelerates wound healing compared to control groups in acute and/or diabetic models. Our work thus offers a multi-target, rationally assembled multicomposite system with strong clinical applicability for chronic diabetic wound management.

## 2. Results and Discussion

### 2.1. Preparation and Characterization of PGAs@CDV

#### 2.1.1. Nanomicelle Construction and Characterization

**Figure 1:**
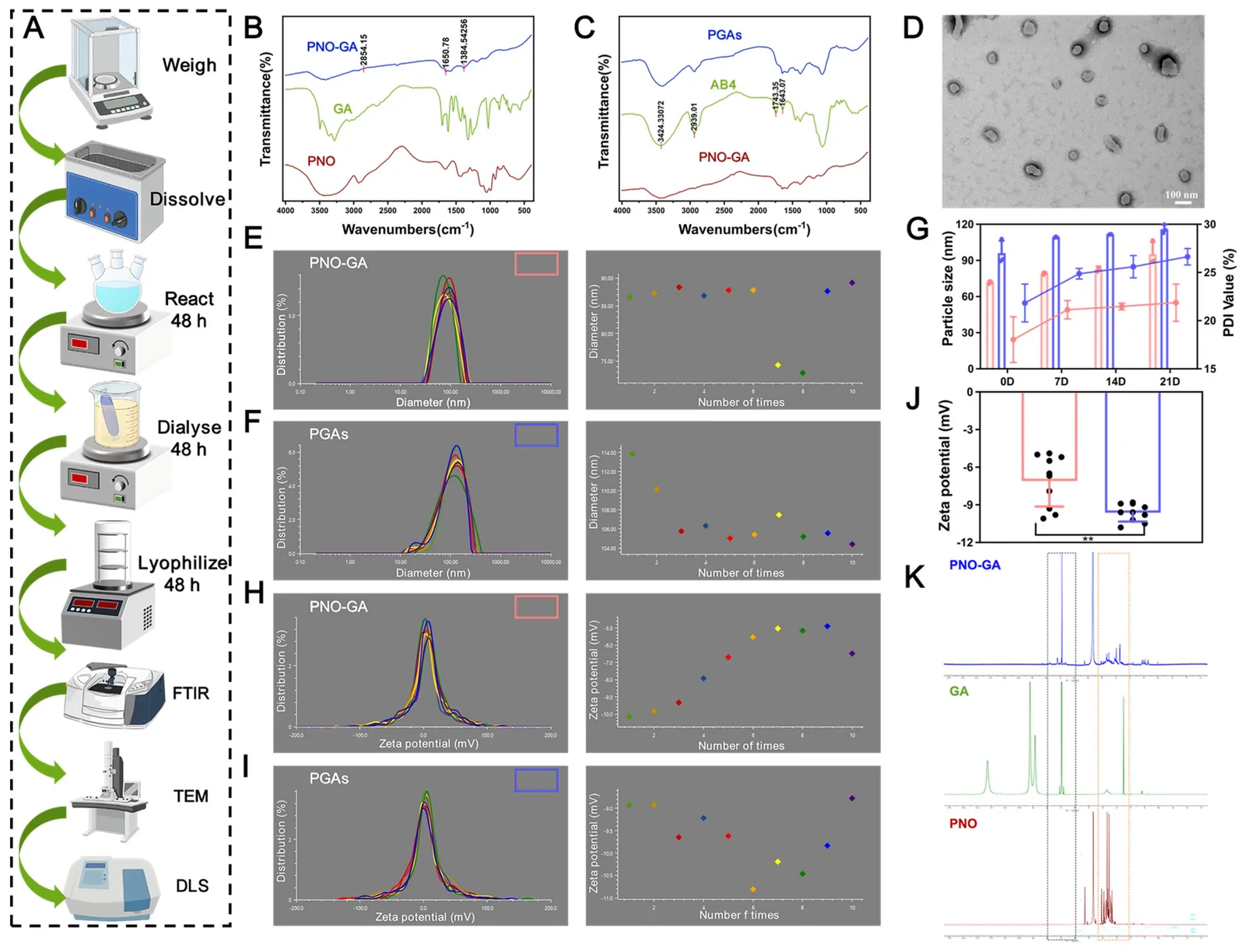
Preparation and characterization of the nanomicelle. (**A**) Schematic of the PNO-GA preparation and characterization workflow. (**B**) FTIR spectra confirming PNO-GA synthesis. (**C**) FTIR spectra confirming AB4 loading in PGAs. (**D**) Representative TEM image of PGAs nanomicelles. (**E**,**F**) Particle size distributions of PNO-GA and PGAs. (**G**) Particle size of PNO-GA and PGAs over 21 days. (**H**−**J**) Zeta potentials of PNO-GA and PGAs. (**K**) Analysis of magnetic resonance imaging. (**E**,**F**,**H**,**I**) Each color represents one independent replicate measurement.Data are presented as mean ± SD; *n* = 10 for (**I**,**J**). Statistical significance was assessed by *t*-test and one-way ANOVA. ** *p* < 0.01.

To optimize AB4 solubility and bioavailability while embedding therapeutic function into the carrier itself, we generated PNO-GA by covalently conjugating skin-repair-active PNO with anti-inflammatory, antioxidant GA. EDC/DMAP-activated carboxyl groups of GA reacted with the hydroxyl-rich PNO to form the conjugate. This conjugate self-assembled via dialysis into amphiphilic nanomicelles with a hydrophobic GA core and hydrophilic PNO shell. AB4 was encapsulated within the hydrophobic core to yield the drug-loaded PGAs nanomicelles. The preparation workflow is illustrated in Figure 1A. FTIR confirmed each synthetic step: the new peak (~1384 cm^−1^) in PNO-GA confirmed covalent GA incorporation, while peak displacement and broadening at ~2854 and ~1650 cm^−1^ indicated hydrogen-bond network formation (Figure 1B). The PGAs spectrum showed superposition of PNO-GA and AB4 features, with the sharpest AB4 characteristic peak markedly attenuated upon encapsulation, confirming AB4 loading into PNO-GA nanoparticles (Figure 1C). TEM imaging of PGAs nanomicelles is shown in Figure 1D. Both PNO-GA and PGAs formed nanoparticles with diameters ranging from 60 to 120 nm, while PGAs displayed a slightly larger average size than PNO-GA. Particle size remained unchanged after 21 days of storage, confirming excellent colloidal stability (Figure 1E–G). Encapsulation of AB4 increased the mean particle diameter by approximately 26 nm, yet particle sizes remained within the optimal range for drug delivery.

Both formulations exhibited narrow size distributions, with PDI values between 0.2 and 0.3. The marginally higher PDI of PGAs (approximately 4%) likely reflects minor variability in AB4 loading but did not compromise overall monodispersity. Zeta potentials ranged from −12 to −3 mV, with PGAs exhibiting a greater absolute value than PNO-GA (Figure 1H–J), consistent with stronger electrostatic repulsion and improved dispersion stability. The increase in particle size likely resulted from expansion of the hydrophobic core following AB4 incorporation, whereas the approximately 3 mV increase in the absolute zeta potential of PGAs probably arose from drug-induced rearrangement of surface polymer chains that exposed additional anionic groups. The ^1^H NMR spectra of PNO, GA, and PNO-GA were then compared. GA showed only the characteristic benzene-ring peaks of gallic acid at δ 6.6–7.2 ppm, with no signals corresponding to polysaccharide sugar rings, whereas PNO displayed dense multiplets from sugar ring protons at δ 2.5–4.1 ppm. The PNO-GA spectrum, by contrast, contained both the aromatic signals of GA and the sugar ring signals of PNO, with characteristic peaks from both starting materials clearly coexisting—preliminary evidence of successful PNO-GA conjugation (Figure 1K).

#### 2.1.2. Hydrogel Construction and Characterization

**Figure 2:**
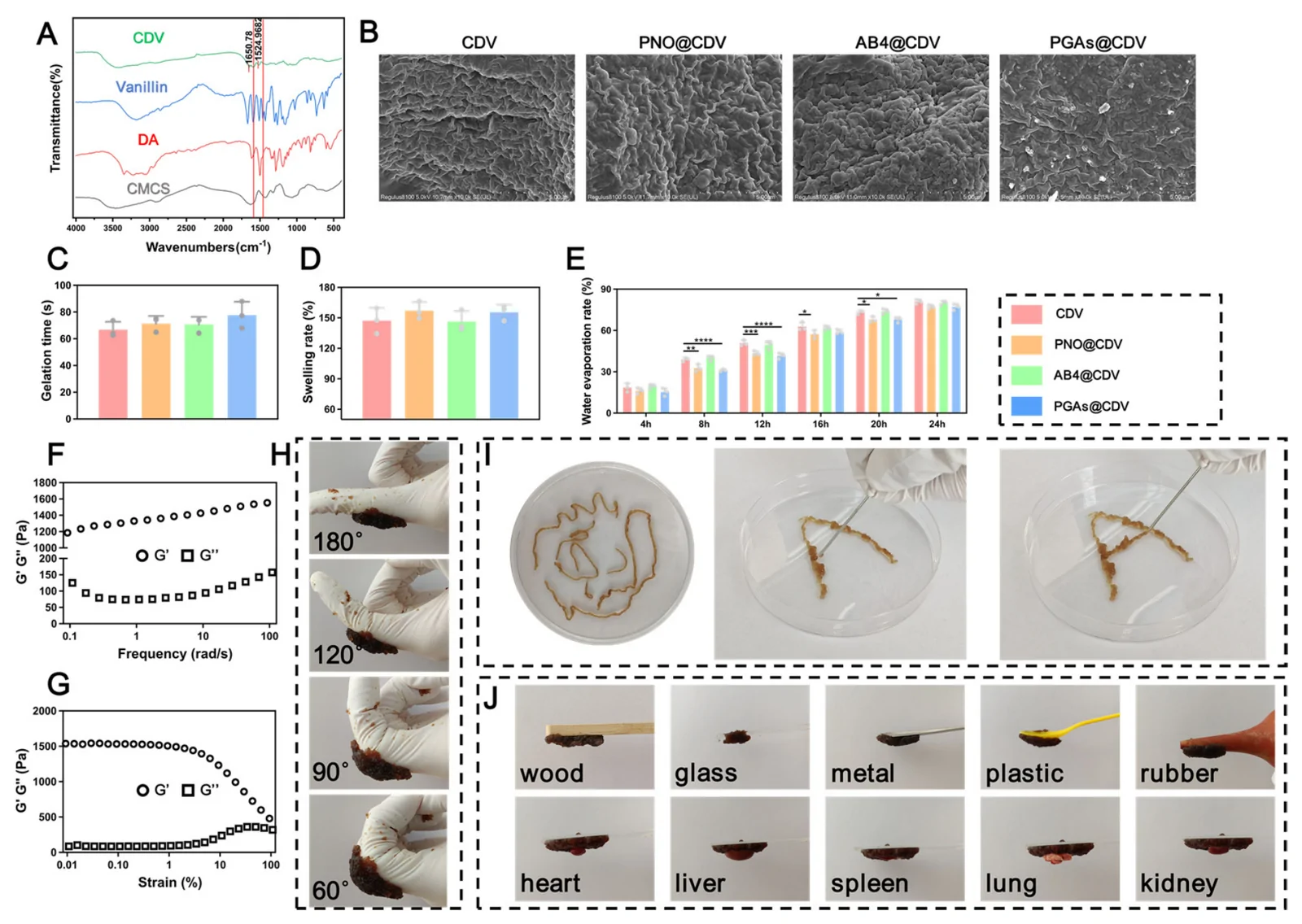
Preparation and characterization of the PGAs@CDV hydrogel system. (**A**) FTIR spectra of the CDV hydrogel. (**B**) SEM images of CDV, PNO@CDV, AB4@CDV, and PGAs@CDV hydrogels. (**C**) Gelation times. (**D**) Swelling ratios of the four hydrogel formulations. (**E**) Water evaporation rates of the four hydrogel formulations. (**F**,**G**) Frequency and strain sweep rheological curves of PGAs@CDV. (**H**) Adhesion of PGAs@CDV to finger joints at varying bending angles (60°, 90°, 120°, 180°). (**I**) Injectability of PGAs@CDV through a 21−gauge needle. (**J**) Adhesion of PGAs@CDV to diverse substrates and biological tissues. Data are presented as mean ± SD; *n* = 3 for (**C**−**E**). Statistical significance was assessed by *t*−test and one-way ANOVA. * *p* < 0.05, ** *p* < 0.01, *** *p* < 0.001, **** *p* < 0.0001.

PGAs nanomicelles were incorporated into a CDV hydrogel dressing to prevent rapid wound-site clearance, extend retention, and sustain drug release. The three-dimensional hydrogel network additionally maintains a moist environment conducive to healing [6]. FTIR analysis of the CDV hydrogel showed disappearance of the vanillin aldehyde peak, formation of a new imine bond peak, and enhanced hydrogen bonding, confirming successful Schiff base crosslinking to form a three-dimensional network (Figure 2A). SEM revealed CDV, PNO@CDV, AB4@CDV, and PGAs@CDV morphology (Figure 2B). Drug loading did not alter gelation time, swelling ratio, or water evaporation rate across all four formulations, indicating that the hydrogel matrix integrity is well preserved (Figure 2C–E). Frequency and strain sweep analyses demonstrated that PGAs@CDV possesses excellent mechanical integrity and structural stability (Figure 2F,G). The hydrogel readily conforms to irregular surfaces (Figure 2H), enabling intimate contact with wounds of varying geometry. It can also be injected through a 21-gauge needle (Figure 2I), allowing minimally invasive administration, and rapidly adheres to diverse biological tissues (Figure 2J), providing stable fixation without the need for sutures. These combined qualities make the hydrogel a versatile and convenient dressing option for surgical procedures.

Diabetic chronic wound healing fails through multifactorial, multistage disruption of the normal wound-healing cascade under hyperglycemic conditions. The four classical phases are each variably impaired in diabetes, but dysregulation of the inflammatory stage is especially consequential. Persistent M1 macrophage dominance and failure to transition to the reparative M2 phenotype establish a state of chronic inflammation that directly suppresses re-epithelialization, angiogenesis, and fibroblast-mediated collagen synthesis in the subsequent proliferative phase [35,36]. We therefore evaluated the hydrogels for multi-target effects in vitro and in vivo.

#### 2.1.3. Drug Release Kinetic Analysis

**Figure 3:**
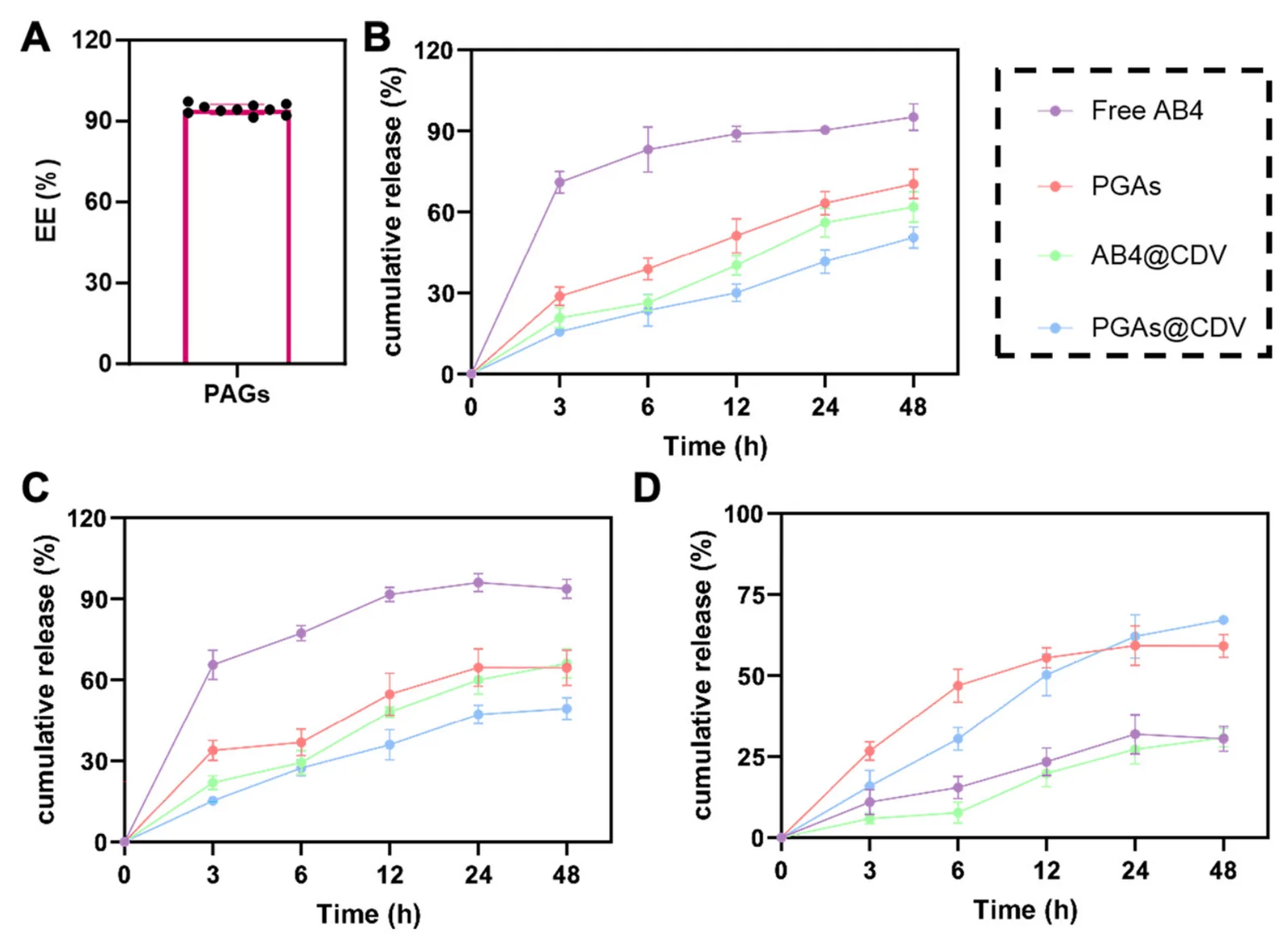
Encapsulation efficiency, in vitro drug release and skin permeation profiles of formulations. (**A**) Encapsulation efficiency of AB4 in PGAs. (**B**) In vitro drug release under standard conditions. (**C**) In vitro drug release under simulated wound conditions. (**D**) In vitro skin permeation. Data are presented as mean ± SD (*n* = 10 for A; *n* = 3 for (**B**–**D**)). Statistical analysis was performed using Student’s *t*-test and one-way ANOVA.

We first characterized the encapsulation efficiency (EE) of AB4-loaded PGAs (Figure 3A): the PGA nanocarriers achieved an EE exceeding 90%, demonstrating excellent loading capacity for AB4. We measured the drug release of each formulation. Under standard conditions (Figure 3B), free AB4 reached about 95% release by 48 h. The three carrier formulations (PGAs, AB4@CDV, and PGAs@CDV) all released more slowly, and PGAs@CDV was the slowest of the three. Dual encapsulation therefore restricted diffusion. The pattern held under simulated wound conditions (Figure 3C), free AB4 released quickly again, and all three carrier formulations sustained release across 48 h. Skin permeation told a similar story (Figure 3D). Free AB4 crossed the skin barrier poorly. PGA-based formulations crossed it much more readily, and PGAs@CDV gave the highest cumulative penetration at 48 h. This last result points to a combined contribution from the PGA nanomicelles and the CDV hydrogel network in transdermal delivery.

Together, the data show two things. First, PGAs load AB4 and carry it as an assembled unit. Second, once the loaded PGAs are dispersed in the CDV hydrogel, drug release slows further and skin penetration goes up. Both effects raise the drug concentration available at the wound site, which is the property that matters for diabetic wound repair.

### 2.2. In Vitro Evaluation Results

#### 2.2.1. Biocompatibility and In Vitro Safety

Hydrogels were soaked in DMEM for 24 h, and the extracts were transferred to cell cultures. This setup approximates how a topical formulation releases material into surrounding tissue in vivo. Three cell types received the extracts: HaCaT keratinocytes, HUVECs, and HDF-a fibroblasts. Each stands in for one of the processes that go wrong in diabetic wound healing: re-epithelialization, blood vessel formation, and extracellular matrix deposition [36,37].

**Figure 4:**
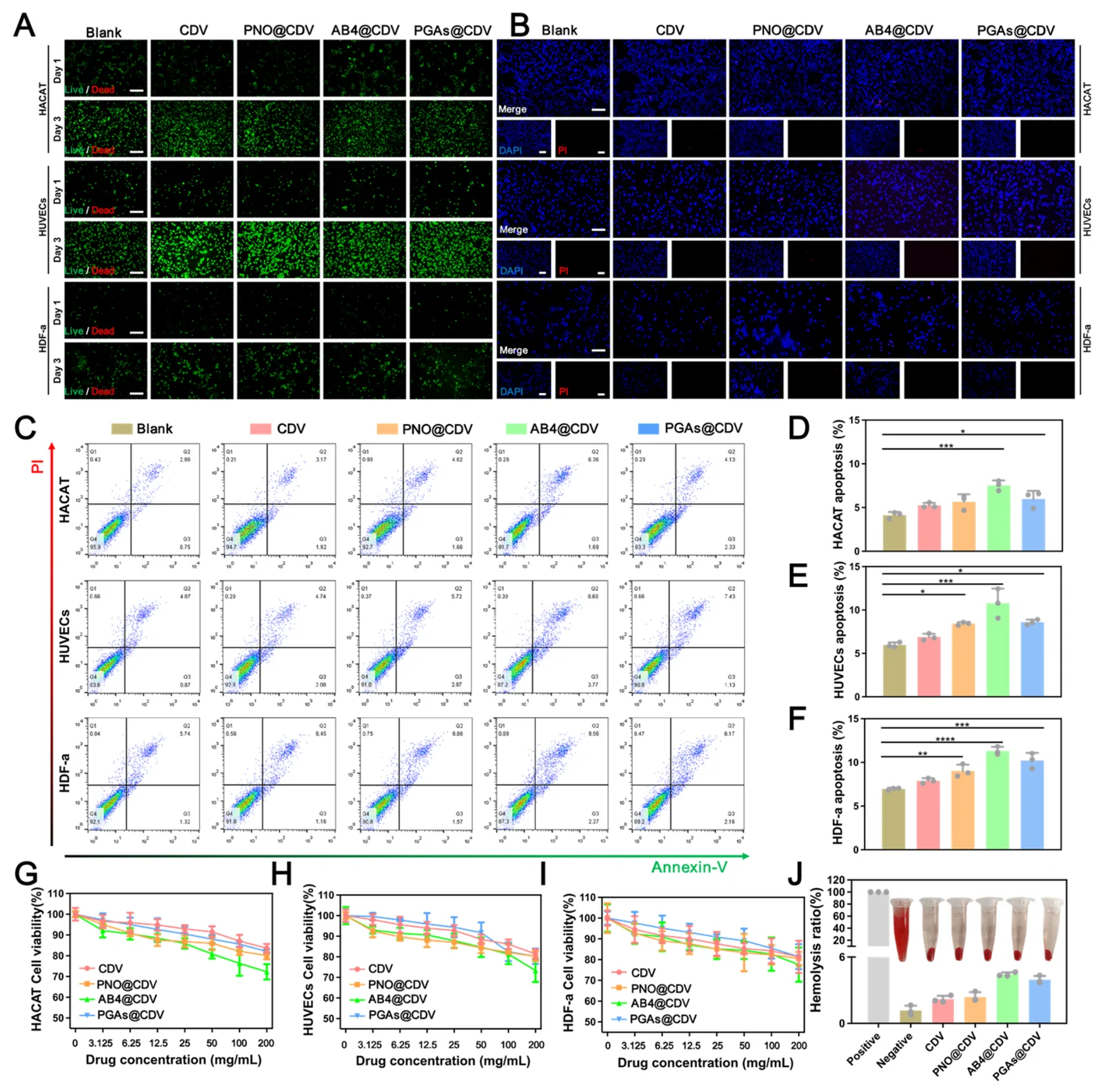
In vitro biocompatibility and safety evaluation. (**A**) Representative live/dead staining images of HaCaT, HUVEC, and HDF-a cells treated with hydrogel extracts. Scale bar = 100 μm. (**B**) Representative fluorescence images after treatment. Scale bar = 100 μm. (**C**) Representative flow cytometry plots. (**D**–**F**) Quantitative apoptosis rates from flow cytometry for HaCaT (**D**), HUVECs (**E**), and HDF-a (**F**) cells. (**G**–**I**) Cell proliferation curves of HaCaT (**G**), HUVECs (**H**), and HDF-a (**I**) cells across serial hydrogel extract concentrations. (**J**) Photographs and quantitative hemolysis rates. Data are mean ± SD; *n* = 3 for (**D**–**F**,**J**), *n* = 5 for (**G**–**I**). Statistical significance was assessed by *t*-test and one-way ANOVA. * *p* < 0.05, ** *p* < 0.01, *** *p* < 0.001, **** *p* < 0.0001.

We ran four biocompatibility assays on CDV, PNO@CDV, AB4@CDV, and PGAs@CDV: live/dead staining (Figure 4A), apoptosis analysis (Figure 4B), flow cytometry (Figure 4C–F), and SRB proliferation across a concentration range (Figure 4G–I). None of the four formulations damaged the three cell types at the concentrations tested. Hemolysis stayed under 5% in every group, and no treatment differed from another (Figure 4J), so hemolytic risk is low. The apoptosis and viability readouts separated the formulations. AB4@CDV had the highest apoptosis and the lowest viability. PGAs@CDV moved in the opposite direction, with less apoptosis and better survival. Loading AB4 into the nanocarrier therefore does two things at once: AB4 becomes more water-soluble, and its cytotoxicity drops.

#### 2.2.2. Cell Migration and Angiogenesis In Vitro

**Figure 5:**
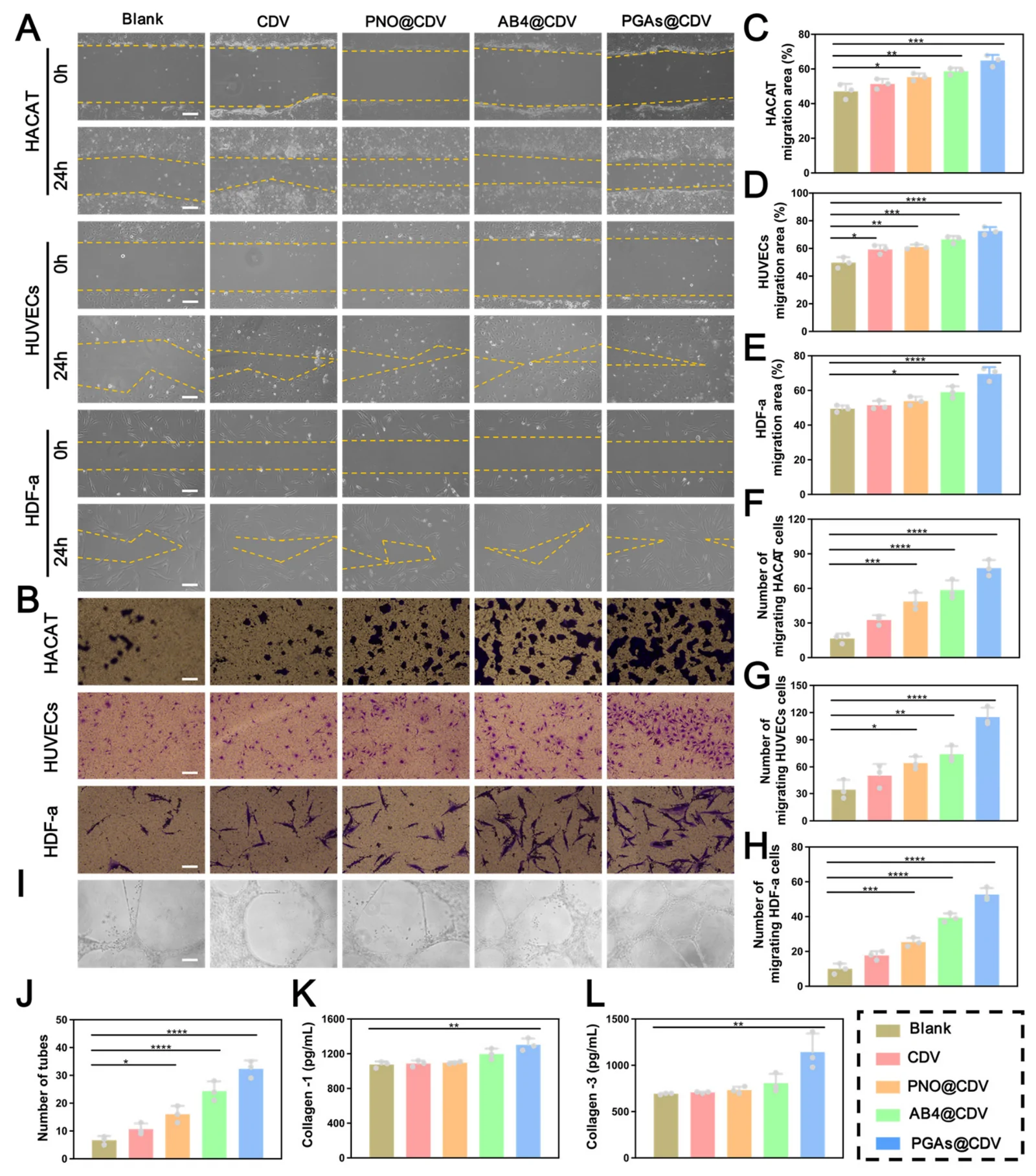
Effects of hydrogel extracts on cell migration, angiogenesis, and collagen deposition. (**A**) Representative scratch assay images of HaCaT, HUVEC, and HDF-a cells. Scale bar = 100 μm. (**B**) Representative Transwell migration images. Scale bar = 100 μm. (**C**–**E**) Quantification of wound closure in HaCaT (**C**), HUVEC (**D**), and HDF-a (**E**) cells. (**F**–**H**) Quantification of migrated cells in Transwell assays for HaCaT (**F**), HUVEC (**G**), and HDF-a (**H**). (**I**) Representative HUVEC tube formation images. Scale bar = 100 μm. (**J**) Quantitative analysis of HUVEC tube formation, including total tube length and junction number. (**K**,**L**) Expression of collagen I (**K**) and collagen III (**L**) in HDF-a cells. Data are presented as mean ± SD (*n* = 3). Statistical analyses were performed using Student’s *t*-test and one-way ANOVA. * *p* < 0.05, ** *p* < 0.01, *** *p* < 0.001, **** *p* < 0.0001.

Scratch wound assays picked up a clear pattern. Extracts from PNO@CDV, AB4@CDV, and PGAs@CDV all sped up migration of HaCaT, HUVEC, and HDF-a cells relative to untreated controls. CDV alone barely moved the needle, with a small uptick in HUVEC migration as the only exception. Transwell gave the same picture: directed migration was higher across the three drug-loaded groups than in controls (Figure 5A–H). These results confirm that the active components, including PNO, AB4, and their composite PGAs, enhance random and directed migration of wound-relevant cell types, a prerequisite for re-epithelialization and granulation tissue formation. Notably, blank CDV selectively promoted HUVEC migration, suggesting intrinsic endothelial compatibility of the biomaterial scaffold and potential indirect activation of endothelial migration pathways, pointing to an inherent pro-angiogenic property of CDV for diabetic wound vascular regeneration. Given that vascular reconstruction is equally essential for wound repair, and that angiogenesis is severely compromised in diabetic wounds, we further assessed tube formation in HUVECs. PNO@CDV-, AB4@CDV-, and PGAs@CDV-treated HUVECs formed markedly denser tubular networks than blank control or CDV groups (Figure 5I,J), demonstrating that the drug-loaded hydrogels also restore endothelial angiogenic function in vitro. Upregulation of HIF-1α, VEGF, CD31, and α-SMA across all four hydrogel groups confirmed that all formulations promote angiogenesis through HIF-1α stabilization and VEGF induction. In contrast, CD31 and α-SMA upregulation provides molecular evidence for new endothelial cell formation and vascular maturation, respectively [36,38].

#### 2.2.3. Collagen Deposition

For collagen synthesis, however, among the tested groups, only PGAs@CDV significantly elevated both type-I and type-III collagen in HDF-a cells (Figure 5K,L), indicating that the combination of all active components is specifically required for ECM restoration. Two things stand out. First, PNO and AB4 each move migration on their own. Second, the assembled PGAs construct outperforms either component alone on angiogenesis and collagen production. Why the assembled construct behaves differently is likely a matter of how the components jointly modulate signaling, but this study does not address that question.

#### 2.2.4. Antioxidant Activity

**Figure 6:**
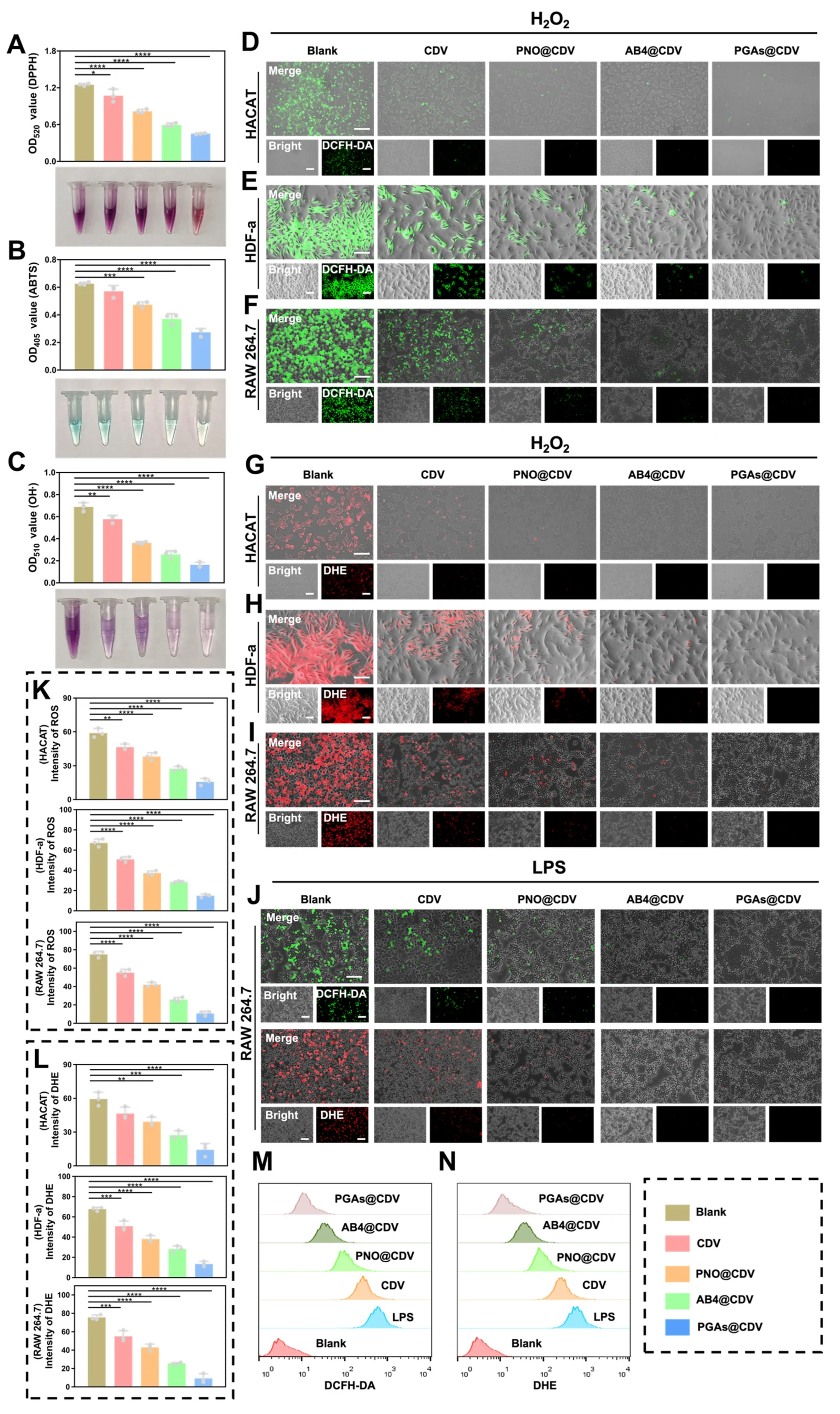
Antioxidant activity of hydrogel extracts in chemical and cellular models. (**A**–**C**) Chemical radical scavenging activity against DPPH (**A**), ABTS^+^ (**B**), and superoxide anions (**C**). (**D**–**F**) Representative fluorescence images of total intracellular ROS (DCFH-DA) in H_2_O_2_-challenged HaCaT (**D**), HDF-a (**E**), and RAW264.7 (**F**) cells after treatment; scale bars = 100 μm (**D**,**E**), 50 μm (**F**). (**G**–**I**) Representative fluorescence images of intracellular superoxide anions (DHE) in H_2_O_2_-challenged HaCaT (**G**), HDF-a (**H**), and RAW264.7 (**I**) cells; scale bars = 100 μm (**G**,**H**), 50 μm (**I**). (**J**) Representative merged fluorescence images (DCFH-DA and DHE) of LPS-stimulated RAW264.7 cells after treatment. Scale bar = 50 μm. (**K**) Quantitative DCFH-DA fluorescence intensity (total ROS) in H_2_O_2_-challenged HaCaT, HDF-a, and RAW264.7 cells. (**L**) Quantitative DHE fluorescence intensity (superoxide anions) in H_2_O_2_-challenged HaCaT, HDF-a, and RAW264.7 cells. (**M**) Flow cytometry quantification of DCFH-DA fluorescence (total ROS) in LPS-stimulated RAW264.7 macrophages. (**N**) Flow cytometry quantification of DHE fluorescence (superoxide anions) in LPS-stimulated RAW264.7 macrophages. Data are presented as mean ± SD (*n* = 3) for (**A**–**C**,**K**,**L**). Statistical significance was assessed by *t*-test and one-way ANOVA. * *p* < 0.05, ** *p* < 0.01, *** *p* < 0.001, **** *p* < 0.0001.

Diabetic wounds sit in a persistently pro-oxidant microenvironment, and that oxidative load is a documented driver of the healing defect [39]. We therefore tested the antioxidant behavior of the hydrogels. Radical scavenging assays put PGAs@CDV ahead of the other formulations on DPPH, ABTS^+^, and superoxide clearance (Figure 6A–C). The cell-based readouts moved in the same direction: in HaCaT, HDF-a, and RAW264.7 cells challenged with H_2_O_2_ or LPS, PGAs@CDV lowered intracellular ROS (DCFH-DA) and superoxide (DHE) more than the other groups (Figure 6D–N). The most plausible reading of these results is compositional. CDV, PNO, and AB4 each carry their own antioxidant activity, and the three of them together give the assembled formulation a broader radical-quenching profile than any component on its own.

#### 2.2.5. Macrophage Polarization and Anti-Inflammatory Activity

Macrophage phenotype is another lever on wound outcomes. In a healthy repair sequence, the pro-inflammatory M1 population (CD86^+^, releasing TNF-α and IL-1β) hands off to the reparative M2 population (CD206^+^, releasing TGF-β and IL-10). Diabetic wounds get stuck: the M1-to-M2 handoff stalls, inflammation lingers, and closure lags [40,41]. To recreate that stall in cell culture, we stimulated RAW264.7 macrophages with LPS. LPS signals through TLR4/NF-κB and pushes the cells toward M1, so CD86 climbs and TNF-α and IL-1β release rises with it [42].

**Figure 7:**
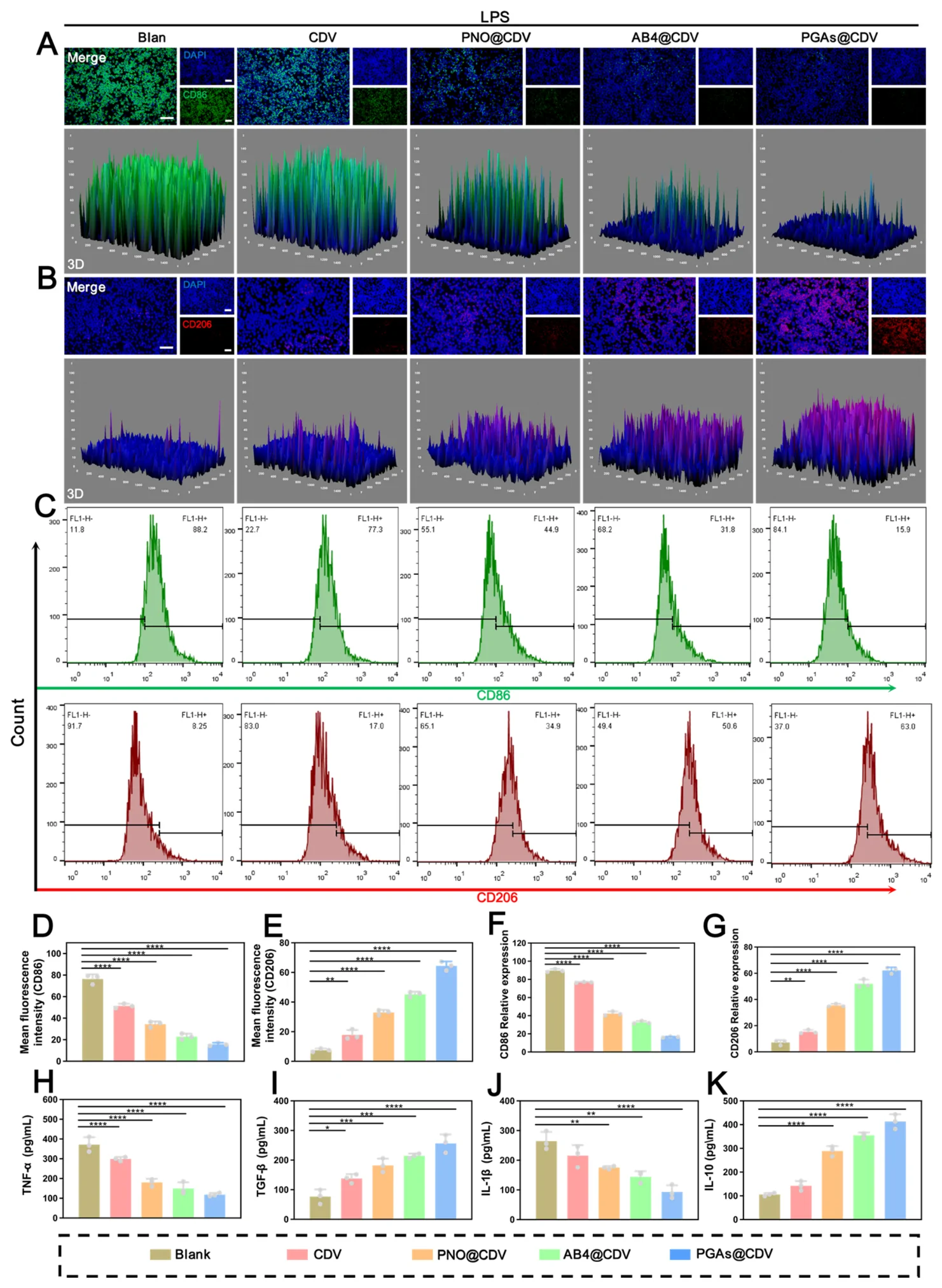
Modulation of macrophage polarization and inflammatory cytokine expression. (**A**,**B**) Representative immunofluorescence for CD86 (**A**) and CD206 (**B**) after treatment. Scale bar = 50 μm. (**C**) Representative flow cytometry plots. (**D**,**E**) Quantitative immunofluorescence analysis of CD86 (**D**) and CD206 (**E**) expression. (**F**,**G**) Quantitative flow cytometry of M1 (**F**) and M2 (**G**) macrophage populations. (**H**–**K**) Quantitative expression of TNF-α (**H**), IL-1β (**I**), TGF-β (**J**), and IL-10 (**K**). Data are mean ± SD (*n* = 3) for (**D**–**K**). Statistical significance was assessed by *t*-test and one-way ANOVA. * *p* < 0.05, ** *p* < 0.01, *** *p* < 0.001, **** *p* < 0.0001.

LPS-stimulated RAW264.7 macrophages responded to all four extracts the same way. CD86, the M1 marker, went down. CD206, the M2 marker, went up. Together these two shifts read as a move away from the pro-inflammatory state and toward the reparative one (Figure 7A–G). PNO@CDV, AB4@CDV, and PGAs@CDV suppressed pro-inflammatory cytokines TNF-α and IL-1β, while CDV alone promoted TGF-β and IL-10 expression without significantly affecting IL-1β or IL-10 (Figure 7H–K). PGAs@CDV suppressed inflammation more than the other three.

### 2.3. In Vivo Evaluation Results

We tested the hydrogels in two wound models: an acute model and an STZ-induced diabetic model. The acute model gives the baseline for how normal tissue heals. The STZ model, by contrast, produces sustained hyperglycemia through selective destruction of pancreatic β-cells, which reproduces the metabolic conditions that make diabetic wounds hard to close [43].

#### 2.3.1. Systemic Biocompatibility

**Figure A1:**
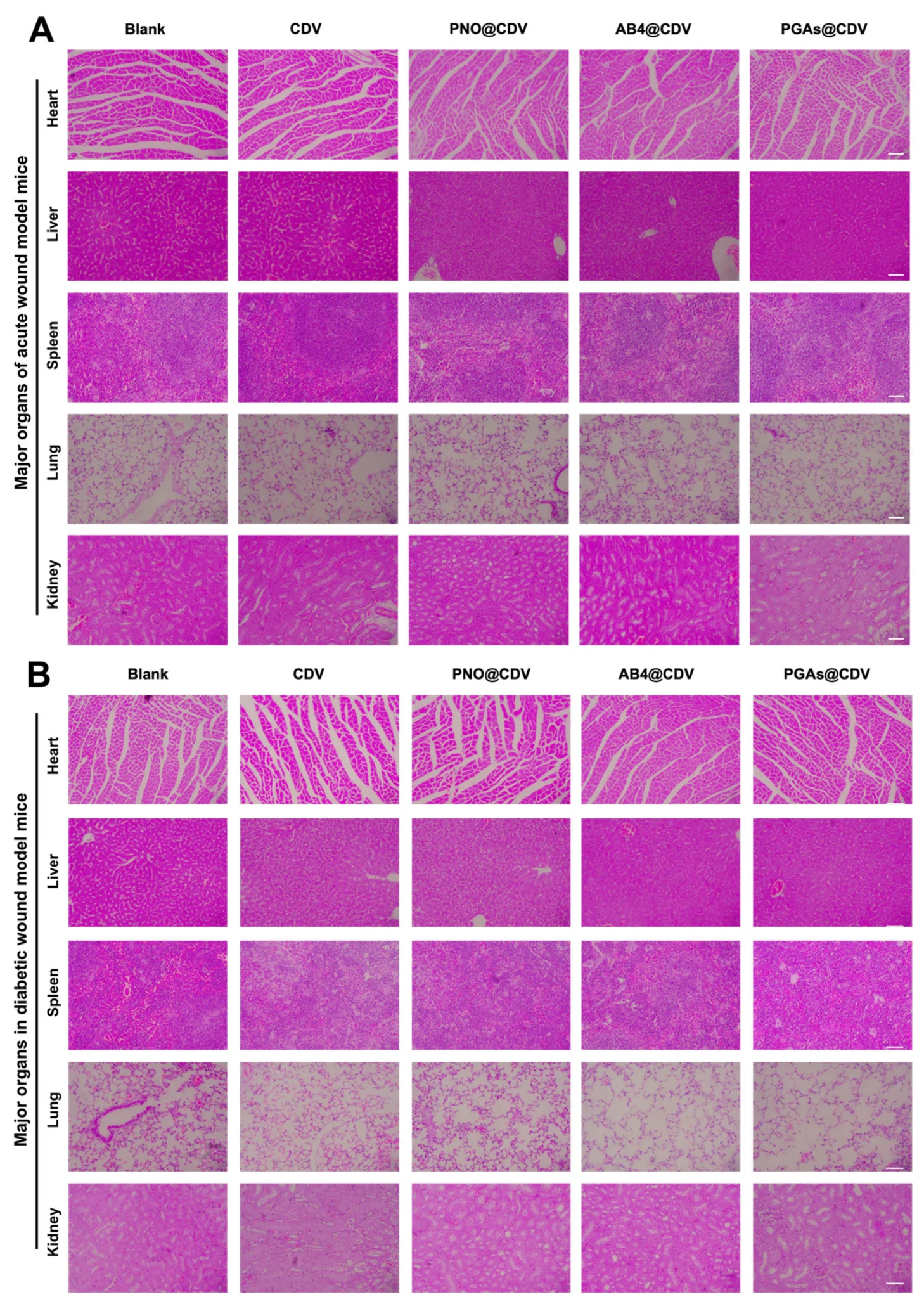
Representative H&E-stained images of major organs from acute (**A**) and diabetic (**B**) wound model mice. Scale bar = 50 μm.

We examined heart, liver, spleen, lungs, and kidneys from both wound models. The examination revealed no detectable pathological abnormalities in either the acute or diabetic wound models. None of the organs showed pathological changes in any treatment group (Figure A1), meaning the hydrogels caused no detectable systemic damage over the study period.

#### 2.3.2. Hemostasis

**Figure 8:**
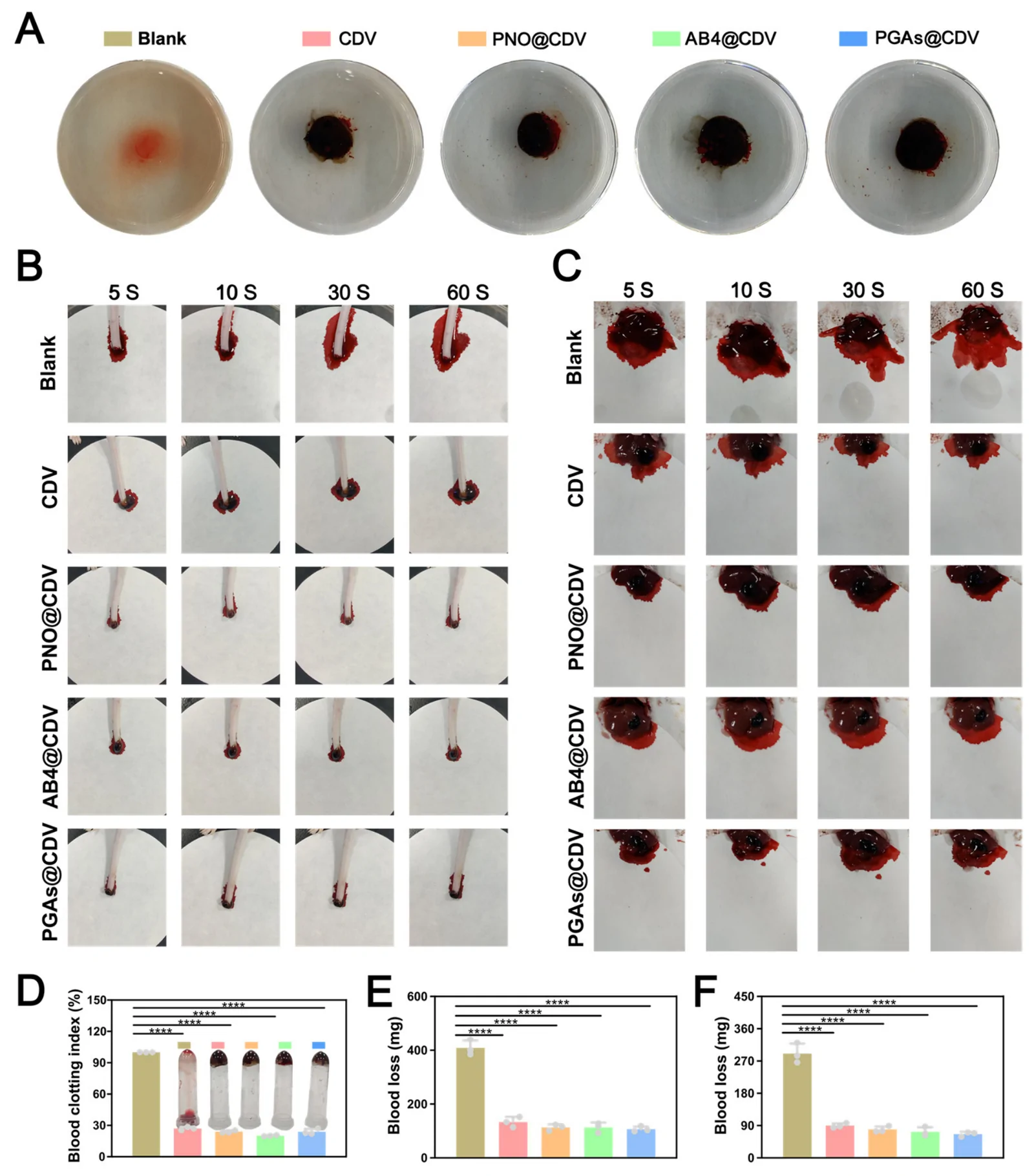
Hemostatic efficacy of hydrogels in mouse tail amputation and liver injury models. (**A**) Representative photographs of in vitro blood coagulation upon direct hydrogel contact. (**B**) Representative photographs of tail amputation hemorrhage and hemostasis after treatment. (**C**) Representative images of liver bleeding before and after treatment. (**D**) Quantitative analysis of in vitro blood coagulation. (**E**,**F**) Quantification of blood loss in the liver injury (**E**) and tail amputation (**F**) models. Data are mean ± SD (*n* = 3); *t*-test and one-way ANOVA. **** *p* < 0.0001.

Diabetes is frequently associated with impaired hemostasis, making rapid bleeding control essential during the early phase of wound management [44]. Chitosan is well-established as a hemostatic biomaterial in wound dressings [45]. In mouse tail amputation and liver injury models, all four hydrogels significantly shortened bleeding time and promoted coagulation relative to untreated controls, without obvious differences among hydrogel groups (Figure 8). This confirms that hemostasis is attributable to the CMCS-containing CDV matrix, which thus serves the dual function of drug carrier and hemostatic scaffold.

#### 2.3.3. Wound Closure

**Figure 9:**
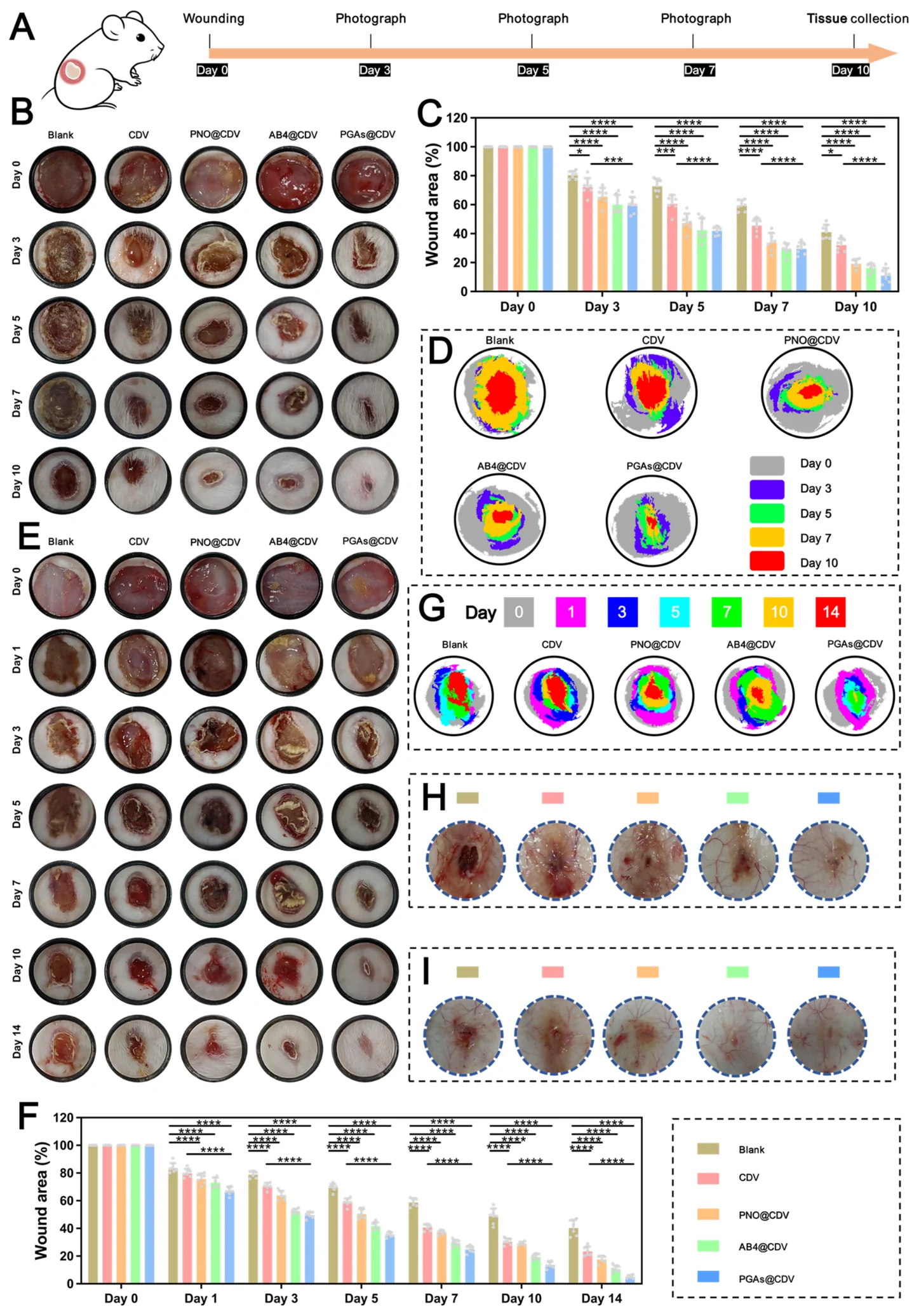
Wound closure and angiogenic effects in acute and diabetic wound models. (**A**) Schematic of the acute wound treatment protocol. (**B**) Representative wound photographs during acute wound treatment. (**C**) Quantitative wound area measurements in the acute model. (**D**) Wound closure trajectory diagrams for the acute model. (**E**) Representative wound photographs during diabetic wound treatment. (**F**) Quantitative wound area measurements in the diabetic model. (**G**) Wound closure trajectory diagrams for the diabetic model. (**H**) Representative subcutaneous vascular photographs at the acute wound site at endpoint. (**I**) Representative subcutaneous vascular photographs at the diabetic wound site at endpoint. Data are presented as mean ± SD (*n* = 6) for (**C**,**F**). Statistical significance was assessed by *t*-test and one-way ANOVA. * *p* < 0.05, *** *p* < 0.001, **** *p* < 0.0001.

Wound closure ran faster in every hydrogel-treated group than in controls across the 10-day acute-model window, and PGAs@CDV closed wounds fastest of the four (Figure 9A–D). Diabetic mice followed the same pattern over 14 days, again with PGAs@CDV in the lead (Figure 9E–G). Subcutaneous vascular imaging at endpoint (day 10 for the acute model, day 14 for the diabetic model) showed denser neovascularization in the hydrogel groups, and the effect was largest under PGAs@CDV (Figure 9H,I).

Why PGAs@CDV outperformed the others is best read as a stacking effect. The formulation touches several repair stages at once: hemostasis is faster, oxidative stress and inflammation are lower, angiogenesis is higher, and collagen deposition is heavier. No single one of these accounts for the overall response; the response tracks with the combination.

#### 2.3.4. Tissue Repair in the Acute Wound Model

**Figure 10:**
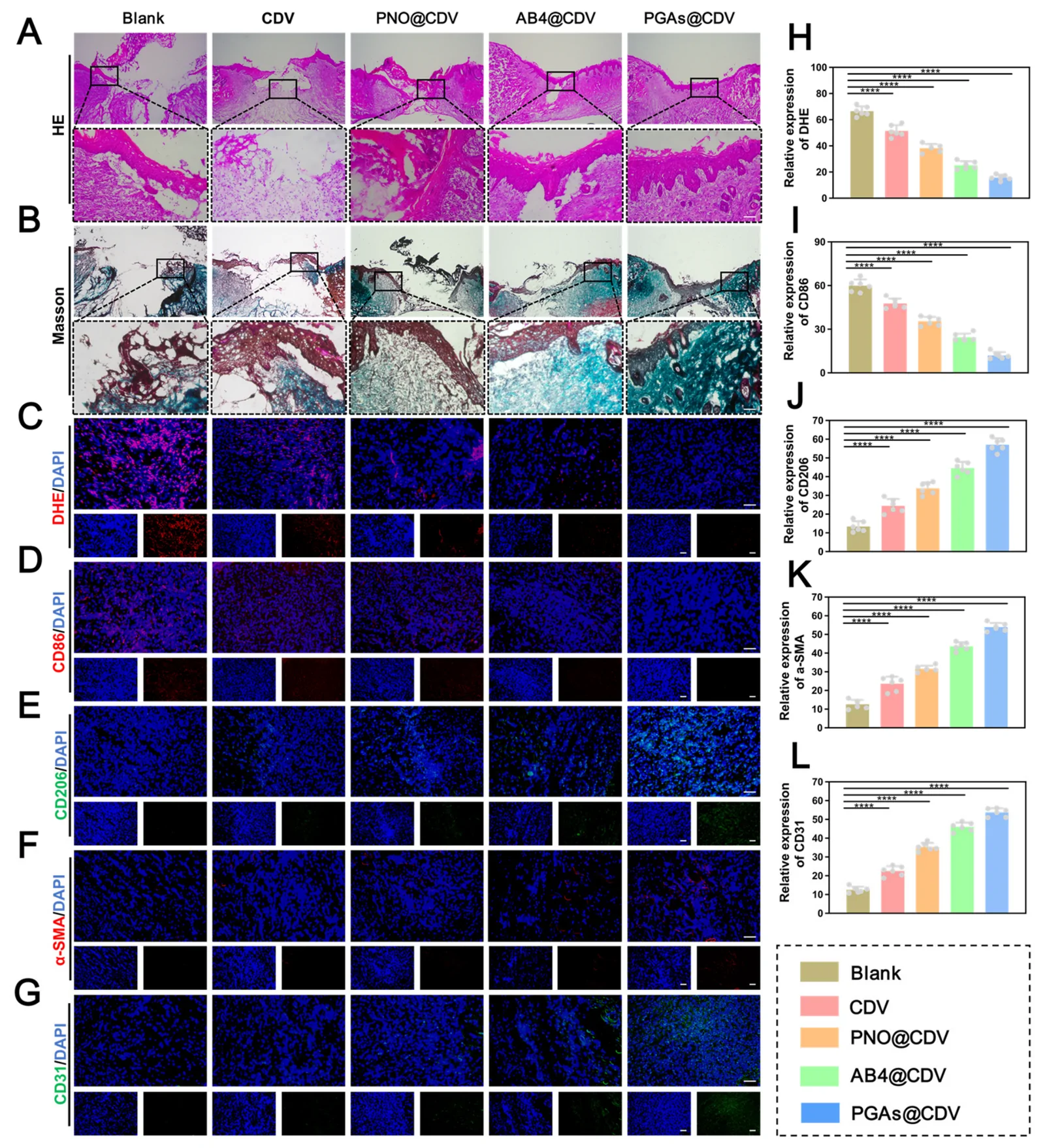
Histological and immunofluorescence analysis of acute wound healing. (**A**) Representative H&E staining images of wound sections. Scale bars = 50 and 250 μm. (**B**) Representative Masson’s trichrome staining images of wound sections. Scale bars = 50 and 250 μm. (**C**–**G**) Representative immunofluorescence images for DHE (**C**), CD86 (**D**), CD206 (**E**), α-SMA (**F**), and CD31 (**G**). Scale bar = 50 μm. (**H**–**L**) Quantitative analysis of DHE (**H**), CD86 (**I**), CD206 (**J**), α-SMA (**K**), and CD31 (**L**) fluorescence intensity. Data are presented as mean ± SD (*n* = 6). Statistical significance was assessed by *t*-test and one-way ANOVA. **** *p* < 0.0001.

Epidermal integrity was assessed on H&E-stained sections by evaluating continuity of the epidermal layer, specifically, whether the individual cell layers remained unbroken and whether any defects, erosion, or ulceration were present. Intact, lesion-free epidermis was classified as having good integrity, while breaks or local defects indicated impaired integrity. Wound defects in the PNO@CDV, AB4@CDV, and PGAs@CDV groups were smaller than in controls, and re-epithelialization covered more of the wound bed. Epidermal architecture recovered further under PGAs@CDV than under either of the other two drug-loaded formulations (Figure 10A). H&E and Masson staining picked up the same ordering. Tissue regeneration progressed across all treatment groups, and the tissue architecture was tidiest in the PGAs@CDV group (Figure 10A,B).

The oxidative and inflammatory readouts moved the same way. DHE staining showed less superoxide in hydrogel-treated wounds, with the largest drop under PGAs@CDV (Figure 10C,H). CD86 (M1) was lower and CD206 (M2) was higher across all hydrogel groups, so macrophage polarization sat closer to the reparative phenotype, and it sat closest under PGAs@CDV (Figure 10D,E,I,J). The vascular markers CD31 and α-SMA both rose after hydrogel treatment, which reads as more angiogenesis and more mature vessels; again, PGAs@CDV led the four groups (Figure 10F,G,K,L).

#### 2.3.5. Tissue Repair in the Diabetic Wound Model

**Figure 11:**
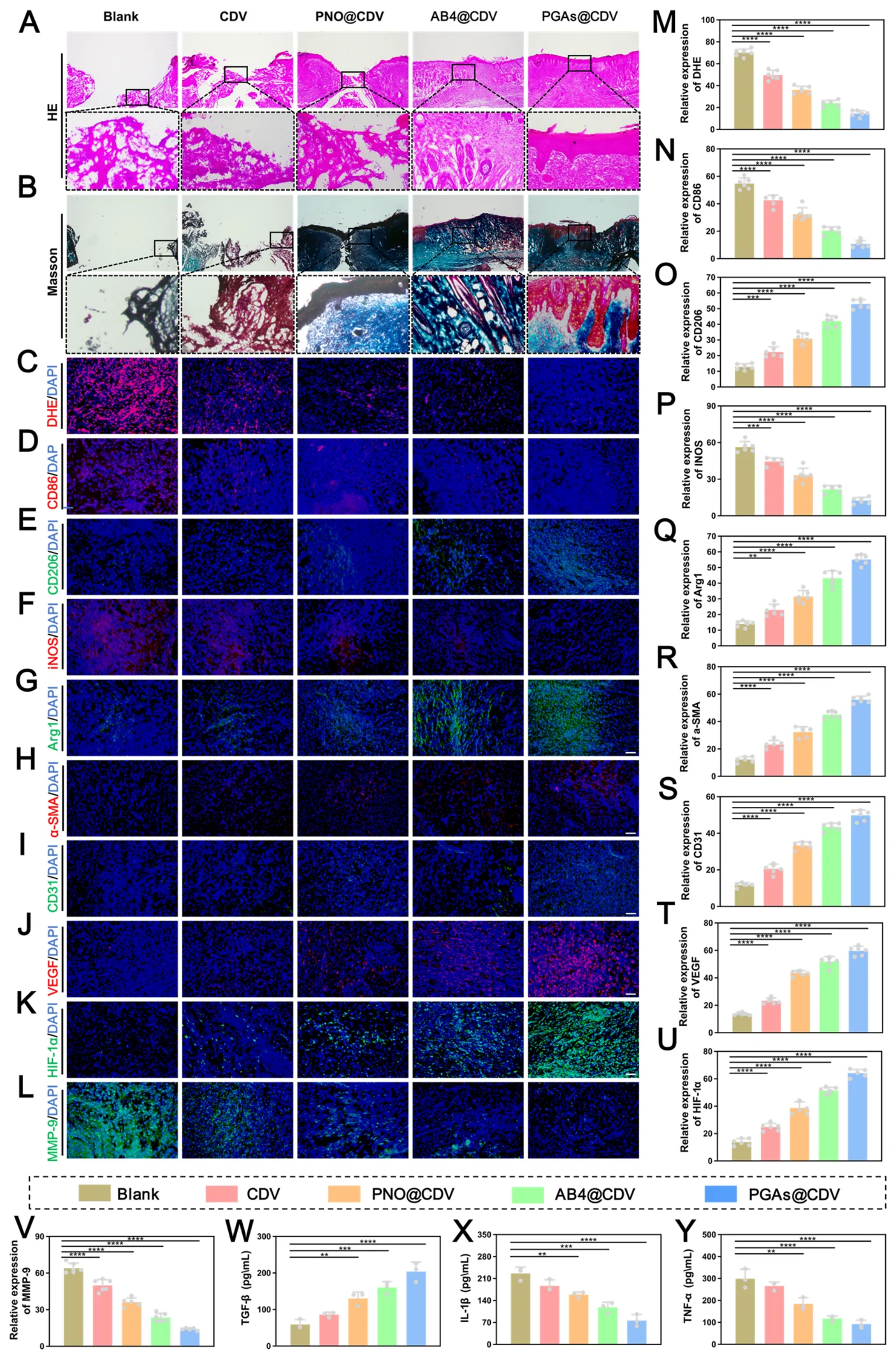
Comprehensive histological and molecular analysis of diabetic wound healing and microenvironment regulation. (**A**) Representative H&E staining of wound sections. Scale bars = 50 and 250 μm. (**B**) Representative Masson’s trichrome staining. Scale bars = 50 and 250 μm. (**C**–**L**) Representative immunofluorescence images for DHE (**C**), CD86 (**D**), CD206 (**E**), iNOS (**F**), Arg-1 (**G**), α-SMA (**H**), CD31 (**I**), VEGF (**J**), HIF-1α (**K**), and MMP-9 (**L**). Scale bar = 50 μm for all panels. (**M**–**V**) Quantitative fluorescence intensity of DHE (**M**), CD86 (**N**), CD206 (**O**), Arg-1 (**Q**), iNOS (**P**), CD31 (**R**), α-SMA (**S**), VEGF (**T**), HIF-1α (**U**), and MMP-9 (**V**). (**W**–**Y**) Quantitative ELISA of TGF-β (**W**), IL-1β (**X**), and TNF-α (**Y**). Data are presented as mean ± SD; *n* = 6 for (**M**–**V**), *n* = 3 for (**W**–**Y**). Statistical significance was evaluated by *t*-test and one-way ANOVA. ** *p* < 0.01, *** *p* < 0.001, **** *p* < 0.0001.

H&E and Masson staining demonstrated the most complete tissue repair in the PGAs@CDV group (Figure 11A,B). DHE levels were reduced in all groups, most markedly with PGAs@CDV (Figure 11C,M). Immunofluorescence confirmed M1-to-M2 polarization across all groups, evidenced by decreased CD86 and increased CD206 and Arg-1 expression, with PGAs@CDV exhibiting the greatest shift (Figure 11D,E,G,N,O,Q). Correspondingly, pro-inflammatory cytokines iNOS, TNF-α, and IL-1β were reduced while TGF-β was elevated in all treatment groups, with PGAs@CDV again performing best (Figure 11F,P,W–Y). Pro-angiogenic markers CD31, α-SMA, VEGF, and HIF-1α were all upregulated, most substantially in the PGAs@CDV group (Figure 11H–K,R–U). Finally, MMP-9 expression, whose overexpression drives ECM degradation and impairs diabetic wound healing [46,47], was strongly lowered in every group, with the greatest suppression in PGAs@CDV (Figure 11L,V). Collectively, these findings demonstrate that PGAs@CDV most effectively attenuates oxidative stress, resolves inflammation, promotes vascular ingrowth, and suppresses ECM degradation, establishing a microenvironment conducive to robust tissue regeneration. In both cellular and animal experiments, anti-inflammatory efficacy ranked CDV < PNO@CDV < AB4@CDV < PGAs@CDV. The activity of blank CDV is attributable to vanillin, a known anti-inflammatory agent [48,49]. PGAs@CDV’s superior performance likely reflects two complementary advantages: nanoscale particle size enhancing cellular uptake and bioavailability, and multicomponent anti-inflammatory activity between PNO and AB4.

CDV is a bioactive matrix. It supports hemostasis, damps inflammation, and promotes endothelial function. Incorporation of PNO primarily enhances cell migration, whereas AB4 contributes potent anti-inflammatory activity. Integrating PNO, GA, and AB4 into a single nanoengineered platform further amplifies these individual effects, producing coordinated antioxidant, anti-inflammatory, pro-angiogenic, and collagen-promoting activities. This design covers the successive stages of wound repair, and this coverage is why PGAs@CDV outperformed the other formulations.

### 2.4. Western Blotting Analysis

Two problems keep diabetic wounds from closing: inflammation that will not resolve, and blood vessels that will not grow back in adequate numbers. Macrophage phenotype sits at the center of the first problem. M1 cells release pro-inflammatory mediators and worsen tissue damage. M2 cells go the other way, they quiet inflammation and support regeneration. NF-κB signaling tips this balance toward M1, and when NF-κB stays on, the inflammatory phase drags out [50]. As inflammation resolves, angiogenesis becomes essential for supplying oxygen and nutrients to regenerating tissue. This process is primarily regulated by the PI3K/AKT-HIF-1α signaling axis, which induces downstream VEGFA and ANG2 expression to stimulate endothelial proliferation and vascular maturation [51]. We ran Western blots for two protein sets: markers of inflammatory regulation, and markers of angiogenesis. The goal was to see what molecular changes tracked with wound repair under PGAs@CDV.

#### 2.4.1. Inflammation-Related Signaling Proteins

**Figure 12:**
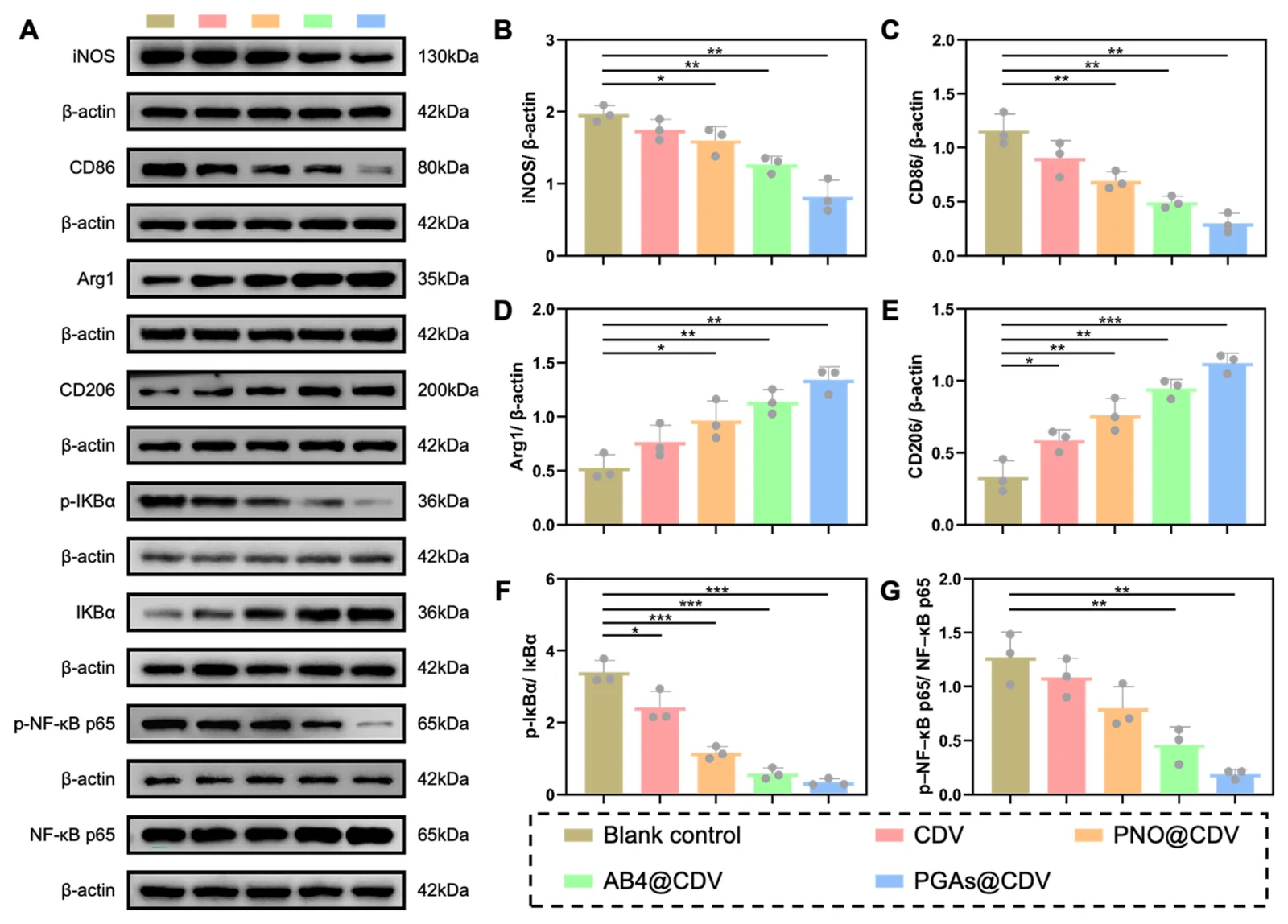
Western blot analysis of macrophage polarization and inflammation-related proteins. (**A**) Representative Western blot bands for iNOS, CD86, Arg1, CD206, p-IκBα, IκBα, p-NF-κB p65, and NF-κB p65. Quantitative analysis of relative expression levels of (**B**) iNOS, (**C**) CD86, (**D**) Arg1, (**E**) CD206, (**F**) p-IκBα/IκBα, and (**G**) p-NF-κB p65/NF-κB p65. Data are presented as mean ± SD; *n* = 3 for (**A**–**G**). Statistical significance was assessed by *t*-test and one-way ANOVA. * *p* < 0.05, ** *p* < 0.01, *** *p* < 0.001.

Western blots for macrophage polarization and NF-κB signaling are shown in Figure 12A. The M1 markers iNOS and CD86 tracked downward from CDV through PNO@CDV and AB4@CDV to PGAs@CDV, and hit their lowest levels in the PGAs@CDV group (Figure 12B,C). The M2 markers Arg1 and CD206 tracked the same gradient in the opposite direction, and PGAs@CDV drove them highest (Figure 12D,E). NF-κB signaling followed suit. Phosphorylated IκBα and phosphorylated NF-κB p65 both dropped across the treated groups, and the drop was largest under PGAs@CDV (Figure 12F,G). These findings indicate that PGAs@CDV suppresses NF-κB activation, promotes macrophage polarization toward the M2 phenotype, reduces the pro-inflammatory M1 population, and thereby establishes a tissue environment favorable for wound regeneration.

#### 2.4.2. Angiogenesis-Related Proteins

**Figure 13:**
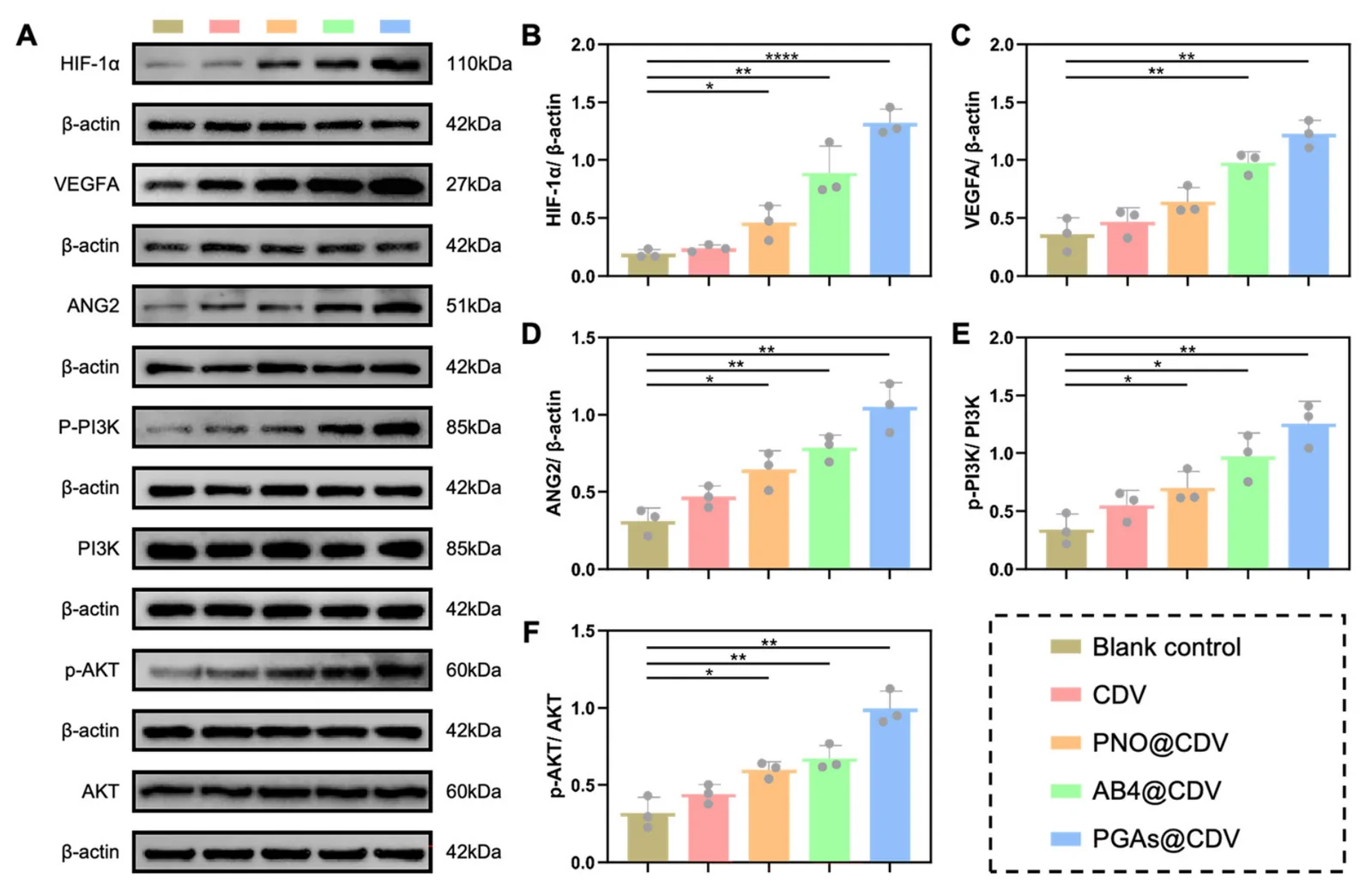
Western blot analysis of angiogenesis-related signaling proteins (**A**) Representative Western blots of HIF-1α, VEGFA, ANG2, p-PI3K, PI3K, p-AKT, and AKT. Quantification of (**B**) HIF-1α, (**C**) VEGFA, (**D**) ANG2, (**E**) p-PI3K/PI3K, and (**F**) p-AKT/AKT. Data are presented as mean ± SD (*n* = 3). Statistical analyses were performed using Student’s *t*-test and one-way ANOVA. * *p* < 0.05, ** *p* < 0.01, **** *p* < 0.0001.

Western blotting was also used to probe the molecular basis of PGAs@CDV-induced angiogenesis (Figure 13A). Relative to the blank control, the CDV hydrogel modestly upregulated HIF-1α, VEGFA, and ANG2, with PNO@CDV and AB4@CDV further increasing these pro-angiogenic proteins; PGAs@CDV produced the highest expression of all three (Figure 13B–D). Activation of the PI3K/AKT cascade followed the same trend, with the phosphorylation ratios of PI3K and AKT rising progressively from blank control through CDV, PNO@CDV, and AB4@CDV to PGAs@CDV, which significantly elevated both p-PI3K and p-AKT levels (Figure 13E,F). Taken together, these findings indicate that PGAs@CDV activates the PI3K/AKT pathway and subsequently upregulates HIF-1α, VEGFA, and ANG2, thereby promoting angiogenesis and supporting wound regeneration.

## 3. Conclusions

We built PGAs@CDV—a nanocomposite hydrogel—for diabetic chronic wounds. PNO was covalently conjugated with GA to give amphiphilic nanomicelles. These carried AB4 and were dispersed in the CDV hydrogel; AB4 became more soluble and more bioavailable. The gel is injectable, biocompatible, and conforms to irregular wounds. Drug loading was high, release sustained, skin permeation greater than that of free AB4, and local retention prolonged. At the pathway level, PGAs@CDV suppressed NF-κB, shifted macrophages from M1 to M2, activated PI3K/AKT/HIF-1α, and raised VEGFA, ANG2, and angiogenesis. In cell and animal work it also scavenged excess ROS, promoted cell migration and collagen synthesis, sped re-epithelialization, and improved tissue regeneration. It outperformed every single-component formulation tested, which supports it as a candidate for diabetic wound treatment.

Several limitations remain. We did not identify the direct molecular targets of the formulation, characterize hydrogel degradation in vivo, or measure drug biodistribution, though CDV bioactivity itself was included in the design and checked against controls. Our models also fell short of the systemic complexity of diabetes. Because this is a proof-of-concept study, we did not benchmark PGAs@CDV against commercial wound dressings, and we did not test whether the covalent conjugation and the multicomponent format add anything beyond the sum of their parts. Follow-up studies with additional control formulations and head-to-head comparisons can close these gaps. Even so, this work lays out a workable design for multifunctional dressings in chronic wound repair.

## 4. Materials and Methods

Carboxymethyl chitosan (CMCS), dopamine hydrochloride (DA), vanillin, EDC, DMAP, DCFH-DA, dihydroethidium (DHE), and DPPH and ABTS^+^ radicals were purchased from Aladdin Biochemical Technology Co., Ltd. (Shanghai, China). Gallic acid (GA) was from Shanghai Macklin Biochemical Co., Ltd. (Shanghai, China). LPS, hematoxylin–eosin (H&E) staining kits, and Annexin V-FITC/PI apoptosis detection kits were from Dalian Meilun Biotechnology Co., Ltd. (Dalian, China). ELISA kits, Masson staining kits were from Solarbio Co., Ltd. (Beijing, China). MMP-9 antibodies were from ABclonal Technology Co., Ltd. (Beijing, China). Anemoside B4 (AB4) was from Chengdu Pufide Biotechnology Co., Ltd. (Chengdu, China). All other reagents were analytical grade.

Recombinant anti-HIF-1αantibody (rabbit mAb), recombinant anti-phospho-PI3K p85 (Y467) + PI3K p55 (Y199) antibody (rabbit mAb), recombinant anti-phospho-AKT (S473) antibody (rabbit mAb), recombinant anti-AKT antibody (rabbit mAb), anti-iNOS/NOS2 antibody, recombinant anti-phospho-IκBα (S32) antibody (rabbit mAb), anti-phospho-NF-κB p65 (S536) rabbit pAb, and anti-NF-κB p65 rabbit pAb were purchased from Wuhan Servicebio Technology Co., Ltd. (Wuhan, China). Anti-VEGF/VEGFA antibody, anti-ANGPT2 antibody, anti-PI3K p85α/PIK3R1 antibody, anti-iNOS/NOS2 antibody, anti-B7-2/CD86 antibody, anti-CD206/MRC1 antibody, and anti-arginase-1/ARG1 antibody were purchased from Wuhan Boster Biological Technology Co., Ltd. (Wuhan, China). 

HaCaT, HUVEC, HDF-a, and RAW264.7 cells were obtained from Procell Biotechnology Co., Ltd. (Wuhan, China). Cells were cultured in DMEM (GIBCO) supplemented with 10% fetal bovine serum (GIBCO) and 1% penicillin-streptomycin (Meilun, Dalian, China) at 37 °C in a humidified 5% CO_2_ atmosphere. Male KM mice (7–8 weeks) were purchased from Liaoning Changsheng Biotechnology Co., Ltd. (Benxi, China). The mice were randomly grouped, and housed under standard conditions (23–26 °C, 40–60% humidity, 12 h light/dark cycle) with free access to food and water. All procedures were conducted in accordance with the Animal Humanities and Ethical Care Committee guidelines of Liaoning University of Traditional Chinese Medicine.

### 4.1. Preparation of PGAs@CDV Hydrogel

#### 4.1.1. Synthesis of Drug-Loaded Nanomicelles (PGAs)

GA, EDC, and DMAP were mixed at 1:1:1 molar ratio to make the activation solution. PNO in DMSO was added dropwise and reacted at 38 °C for 48 h. The mixture was precipitated with ten volumes of ice-cold anhydrous ethanol, held overnight, filtered, washed sequentially with anhydrous ethanol and diethyl ether, and dried to give PNO-GA. PNO-GA was redissolved in DMSO, sonicated, and dialyzed against distilled water for 48 h to give blank micelles. AB4 in ethanol was sonicated, added dropwise to the blank micelles under stirring, and incubated for 2 h. Ethanol was removed by rotary evaporation, and the suspension was diluted with distilled water to give the PGAs nanomicelle solution (PNO, 1 mg/mL; AB4, 0.1 mg/mL).

#### 4.1.2. Characterization of the Nanomicelles

Chemical structures were characterized by FTIR spectroscopy (Shimadzu 8300, Kyoto, Japan) scanning 4000–400 cm^−1^ at 4 cm^−1^ resolution. Hydrodynamic diameter, PDI, and zeta potential were measured by dynamic light scattering (Litesizer 500, Anton Paar, Graz, Austria). Data are mean ± SD from three independent measurements.

#### 4.1.3. Hydrogel Preparation

CMCS, DA, and vanillin were used at a molar ratio of 2:1:1. CMCS and DA were dissolved in deionized water. Vanillin was dissolved at 50 °C. The three solutions were mixed, stirred for 30 min, and left undisturbed for an additional 30 min to form the blank CDV hydrogel. PGAs@CDV, PNO@CDV, and AB4@CDV were prepared by adding PGAs nanomicelles, PNO solution (1 mg/mL), or AB4 solution (0.1 mg/mL) to the hydrogel precursor at 1:50 (*v*/*v*) before gelation.

#### 4.1.4. Hydrogel Characterization

Gelation time was defined as the time for a magnetic stir bar rotating at 100 rpm to stop within the precursor solution. Hydrogel morphology was examined by SEM-EDS (Hitachi Regulus 8100, Hitachi High-Tech, Hitachinaka, Japan) after freeze-drying for 48 h and gold sputter coating. Swelling ratio was determined by immersing freeze-dried samples (W_0_) in PBS at 37 °C and calculated as SR (%) = [(W_t_ − W_0_)/W_0_] × 100%. Water retention was assessed by weighing samples every 4 h during incubation at 50 °C for 24 h, with evaporation rate calculated as [(W − W_0_)/W_0_] × 100%. Adhesion was tested by applying hydrogels to substrates bent at 60°, 90°, 120°, and 180° and recording detachment. Injectability was tested by extrusion through a 21-gauge needle using a 10 mL syringe. Rheology was measured on a rotational rheometer (MCR302e, Anton Paar, Graz, Austria) in frequency- and strain-sweep modes.

#### 4.1.5. Drug Release Kinetic Assays

Drug release was evaluated using dialysis bags containing 1 mL of free AB4, AB4@CDV, PGAs, or PGAs@CDV. Each bag was immersed in 10 mL PBS (pH 7.4) maintained at 35 ± 5 °C with continuous stirring at 100 rpm. At 3, 6, 12, 24, and 48 h, 1 mL of release medium was collected and replaced with fresh PBS. AB4 concentrations were quantified by HPLC (Agilent 1100 Series, Agilent Technologies, Santa Clara, CA, USA), and cumulative release profiles were calculated.

To simulate the acidic wound environment, the same protocol was performed in PBS (pH 5.5). The lower pH reproduces the acidic microenvironment associated with impaired perfusion, elevated metabolic demand, and accumulation of acidic metabolites in chronic wounds. Drug concentrations at each sampling point were determined by HPLC to generate release curves under simulated wound conditions.

For skin permeation studies, dorsal skin harvested from mice was carefully excised after hair removal, cleaned of subcutaneous tissue, rinsed with PBS, inspected microscopically to confirm tissue integrity, and stored at 4 °C until use. Skin permeation was evaluated using Franz diffusion cells with 1 mL of each formulation applied to the donor chamber. Samples of receptor fluid were collected at the designated time points, replaced with fresh PBS, and analyzed by HPLC to determine cumulative transdermal permeation.

Drug release was measured using dialysis bags containing 1 mL of free AB4, AB4@CDV, PGAs, or PGAs@CDV. Each bag was placed in 10 mL PBS (pH 7.4) at 35 ± 5 °C with stirring at 100 rpm. At 3, 6, 12, 24, and 48 h, 1 mL of medium was collected and replaced with fresh PBS. AB4 was quantified by HPLC (Agilent 1100 Series), and cumulative release was calculated.

To simulate the acidic wound environment, the same protocol was run in PBS (pH 5.5). This pH reflects the acidic microenvironment of chronic wounds, driven by impaired perfusion, high metabolic demand, and accumulation of acidic metabolites. AB4 at each time point was quantified by HPLC to generate release curves.

For skin permeation, dorsal skin was excised from mice after hair removal, cleared of subcutaneous tissue, rinsed with PBS, inspected under a microscope for integrity, and stored at 4 °C until use. Permeation was measured in Franz diffusion cells with 1 mL of each formulation in the donor chamber. Receptor fluid was collected at the specified time points, replaced with fresh PBS, and analyzed by HPLC to determine cumulative transdermal permeation.

### 4.2. In Vitro Evaluation

CDV, PNO@CDV, AB4@CDV, and PGAs@CDV hydrogels were soaked in DMEM at 1:2 (hydrogel:medium, *w*/*v*) for 24 h. Extracts were filtered and added to cell cultures at 10% of the final culture for all subsequent in vitro experiments.

#### 4.2.1. In Vitro Safety Evaluation

For live/dead analysis, HaCaT, HUVEC, and HDF-a cells were seeded at 1.5 × 10^4^ cells per well and cultured with hydrogel extracts for 48 h. Cells were then washed with PBS, stained with calcein-AM (1 mM) and propidium iodide (2.5 mg/mL) for 30 min, and imaged using a fluorescence microscope (Nikon Eclipse E800, Nikon Corporation, Tokyo, Japan). Apoptosis was assessed by Annexin V-FITC/PI double staining in cells (1.5 × 10^5^/well) treated for 48 h and analyzed by flow cytometry (BD Biosciences, Franklin Lakes, NJ, USA). Cytotoxicity was evaluated by the sulforhodamine B (SRB) assay: cells (1.5 × 10^4^/well) were treated for 48 h, fixed with 10% cold trichloroacetic acid at 4 °C for 1 h, stained with 0.4% SRB for 20 min, washed with 1% acetic acid, and solubilized in 10 mmol/L Tris base. Absorbance was measured at 540 nm (HBS-1096A, DB, Nanjing, China); survival rate (%) = (A_1_/A_0_) × 100%. For hemolysis, a 2% (*v*/*v*) mouse RBC suspension was mixed with hydrogel extracts and incubated at 37 °C for 2 h. Purified water and saline served as positive and negative controls, respectively. Supernatant absorbance was measured at 540 nm, and the hemolysis rate (HR) was calculated as: HR (%) = [(Ah − An)/(At − An)] × 100%.

#### 4.2.2. Evaluation of In Vitro Efficacy

Scratch wound assays were performed in 6-well plates (1.5 × 10^5^ cells/well). After 12 h of culture, uniform scratches were made, detached cells were removed by PBS washes, serum-free medium containing hydrogel extracts was added, and scratch width was imaged at 0 and 24 h (Nikon Eclipse E800). For Transwell migration, cells (1 × 10^4^ in 100 μL serum-free medium) were seeded in the upper chamber with hydrogel extracts; the lower chamber contained 600 μL complete medium. After 48 h, migrated cells were fixed with 4% paraformaldehyde and stained with crystal violet.

Tube formation assay. Matrigel (50 μL/well) was thawed on ice overnight and polymerized at 37 °C for 30 min. HUVECs (2 × 10^4^/well) were seeded onto Matrigel and co-cultured with each hydrogel extract. Tube formation was photographed and analyzed.

Collagen Deposition. HDF-a cells (2.5 × 10^5^/well) were scratch-wounded after 12 h of culture, washed with PBS, and co-cultured with hydrogel extracts in serum-free medium for 48 h. Cell lysate supernatants were collected. Collagen-I and Collagen-III levels were quantified by ELISA per the manufacturer’s instructions.

Antioxidant Activity. DPPH scavenging was measured after 30 min of dark incubation at 517 nm. ABTS^+^ scavenging was measured at 734 nm after 6 min. Superoxide anion scavenging was assessed chemically via the catechol autoxidation system in Tris-HCl buffer (pH 8.2) at 325 nm over 4 min, and cellularly using DHE (5 μM) in H_2_O_2_- or LPS-pretreated cells by fluorescence microscopy (Ex/Em = 518/605 nm) and flow cytometry. Total intracellular ROS was measured using DCFH-DA (10 μM) in H_2_O_2_- or LPS-treated cells at 37 °C for 30 min (Ex/Em = 488/525 nm).

Anti-inflammatory and macrophage polarization. RAW264.7 cells (1 × 10^5^/well) were cultured for 12 h, stimulated with LPS for 24 h, then co-cultured with hydrogel extracts. M1 (CD86) and M2 (CD206) polarization markers were assessed by immunofluorescence and flow cytometry. Secreted TNF-α, IL-1β, TGF-β, and IL-10 in culture supernatants were quantified by ELISA.

### 4.3. In Vivo Evaluation

#### 4.3.1. Animal Models

For the acute wound model, mice were anesthetized with 4% pentobarbital, depilated, and full-thickness 8 mm circular wounds were created with a biopsy punch. For the diabetic wound model, diabetes was induced by a two-week STZ injection. Blood glucose levels were randomly monitored in a subset of STZ-injected mice, and only those with levels consistently exceeding 16 mmol/L were used for subsequent wounding experiments. Diabetic mice were anesthetized, and wounds were created as above. All animals were acclimatized in SPF facilities for two weeks prior to experimentation.

#### 4.3.2. Safety Assessment

Heart, liver, spleen, lung, and kidney were harvested post-treatment and assessed by H&E staining for histopathological changes.

#### 4.3.3. Animal Efficacy Evaluation

For liver hemostasis, anesthetized mice were fixed at 30°, the liver was surgically exposed, and a wound was created with a 20-gauge needle. One gram of hydrogel was applied immediately, and blood absorbed by pre-weighed filter paper was photographed and recorded gravimetrically. For the tail hemostasis model, one-third of the tail was amputated under anesthesia, pre-weighed filter paper was placed below the wound, and blood loss was recorded. For in vitro hemostasis, 0.5 g of each hydrogel was pre-warmed at 37 °C for 5 min, 50 μL of anticoagulated whole blood was added, incubated for a further 5 min, and photographed. The blood coagulation index (BCI) was determined by adding 5 mL deionized water to the samples, incubating for 5 min, and reading supernatant absorbance at 540 nm.

In the acute wound model, wound healing was documented by digital photography on days 0, 3, 5, 7, and 10 after injury, and wound areas were quantified using ImageJ software (version 1.54g, National Institutes of Health, USA). At day 10, the subcutaneous vascular network was imaged before wound tissues were harvested, fixed in 4% paraformaldehyde, and processed for H&E staining, Masson’s trichrome staining, and immunofluorescence analysis of CD86/CD206, CD31, and DHE. In the diabetic wound model, wounds were photographed on days 0, 1, 3, 5, 7, 10, and 14. Tissue sections were subsequently analyzed by H&E staining, Masson’s trichrome staining, and immunofluorescence for CD86/CD206, DHE, MMP-9, CD31, α-SMA, VEGF, HIF-1α, and Arg-1. Tissue levels of TNF-α, iNOS, IL-1β, and TGF-β were determined by ELISA.

### 4.4. Detection of Target Proteins by Western Blotting

Total protein was extracted from wound tissues in lysis buffer and quantified by BCA assay. Samples were denatured at 100 °C for 10 min, separated by SDS-PAGE, and transferred to PVDF membranes. Membranes were blocked in 5% non-fat milk for 1 h, then incubated overnight with primary antibodies against PI3K, p-PI3K, AKT, p-AKT, HIF-1α, VEGFA, ANG2, IκBα, p-IκBα, NF-κB p65, p-NF-κB p65, iNOS, CD86, Arg1, and CD206. Following washing, membranes were incubated with corresponding secondary antibodies for 2 h. β-Actin was the loading control, and band intensities were quantified in ImageJ.

### 4.5. Statistical Analysis

Data are presented as mean ± SD. Statistical analyses were performed using GraphPad Prism 9.0. Differences among groups were assessed by one-way ANOVA followed by the Student–Newman–Keuls post hoc test. *p* < 0.05 represents statistical significance.

## Data Availability

The original contributions presented in this study are included in the article. Further inquiries can be directed to the corresponding authors.

## Abbreviations

The following abbreviations are used in this manuscript:

AB4	Anemoside B4
ABTS^+^	2,2′-Azino-bis(3-ethylbenzothiazoline-6-sulfonic acid) radical cation
AKT	Protein Kinase B (PKB)
α-SMA	Alpha-Smooth Muscle Actin
ANG2	Angiopoietin-2
ANOVA	One-way Analysis of Variance
Arg-1	Arginase-1
BCI	Blood Coagulation Index
CDV	Carboxymethyl chitosan-dopamine-vanillin hydrogel
CD31	Platelet Endothelial Cell Adhesion Molecule-1
CD86	Cluster of Differentiation 86
CD206	Cluster of Differentiation 206
CMCS	Carboxymethyl Chitosan
DA	Dopamine Hydrochloride
DCFH-DA	2′,7′-Dichlorodihydrofluorescein Diacetate
DHE	Dihydroethidium
DMSO	Dimethyl Sulfoxide
DMAP	4-Dimethylaminopyridine
DMEM	Dulbecco’s Modified Eagle Medium
DPPH	1,1-Diphenyl-2-picrylhydrazyl
EDC	1-Ethyl-3-(3-dimethylaminopropyl) carbodiimide
ELISA	Enzyme-Linked Immunosorbent Assay
FITC	Fluorescein Isothiocyanate
GA	Gallic Acid
H&E	Hematoxylin and Eosin
HIF-1α	Hypoxia-Inducible Factor 1 Alpha
HUVECs	Human Umbilical Vein Endothelial Cells
HaCaT	Human Keratinocyte Cell Line
HDF-a	Human Dermal Fibroblasts-Adult
HR	Hemolysis Rate
IHME	Institute for Health Metrics and Evaluation
IL-1β	Interleukin-1 Beta
IL-10	Interleukin-10
iNOS	Inducible Nitric Oxide Synthase
IκBα	Nuclear factor of kappa light polypeptide gene enhancer in B-cells inhibitor, alpha
LPS	Lipopolysaccharide
MMP	Matrix Metalloproteinase
MMP-9	Matrix Metalloproteinase-9
NF-κB p65	Nuclear factor kappa-B subunit p65 (RelA)
PDI	Polydispersity Index
PEG	Polyethylene Glycol;
PNO	*Panax notoginseng* Oligosaccharide
PNO-GA	Amphiphilic polymer micelles of PNO and GA
PGAs	PNO-GA micelles loaded with Anemoside B4
PGAs@CDV	PGAs nanomicelles embedded in CDV hydrogel
p-AKT	Phosphorylated AKT
PI	Propidium Iodide
p-IκBα	Phosphorylated IκBα
p-NF-κB p65	Phosphorylated NF-κB p65
pHPMA	N-(2-Hydroxypropyl) methacrylamide
p-PI3K	Phosphorylated phosphatidylinositol 3-kinase
PVP	Poly(N-vinyl-2-pyrrolidone)
PI3K	Phosphatidylinositol 3-kinase
RAW 264.7	Mouse Leukemic Monocyte Macrophage Cell Line
RBCs	Red Blood Cells
ROS	Reactive Oxygen Species
SD	Standard Deviation
SEM	Scanning Electron Microscopy
SPF	Specific Pathogen-Free
SR	Swelling Ratio
SRB	Sulforhodamine B
STZ	Streptozotocin
TEM	Transmission Electron Microscopy
TGF-β	Transforming Growth Factor Beta
TNF-α	Tumor Necrosis Factor Alpha
VEGF	Vascular Endothelial Growth Factor
VEGFA	Vascular Endothelial Growth Factor A

## Appendix A

**Table A1:** Summary of reported properties of representative hydrogel dressings.

Property	PGAs@CDV (This Work)	GelMA@FcS [31]	INS@PL-PVA/DOP-CaCO_3_ [32]	FVP@CCN [33]	Ber@CF Gel [34]
Hydrogel Type	Drug-loaded nanomicelle composite hydrogel	Synthetic polymer hydrogel	Polysaccharide-based hydrogel	Polysaccharide-based nanozyme hydrogel	oxidation-reactive supramolecular hydrogel
Gelation Time	~80 s	Gelation observed; no quantitative gelation time reported	N/A	gelation observed; no quantitative gelation time reported	N/A
Swelling Ratio (%)	~150%	~800%	~500%	~600–1200%	N/A
Mechanical properties	G’ > G″ across frequency sweep, demonstrating stable elastic behavior	Compression strength ~20 kPa, elastic hydrogel	G’ > G″ across frequency sweep, indicating stable elastic gel	Compression strength ~20 kPa, elastic hydrogel	G’ > G″ across frequency sweep, indicating stable elastic gel
In vivo Wound Closure Time (Diabetic Model, to ~90% Closure)	~14 days	~10 days	~14 days	~10 days	~14 days

Note: Values are compiled from independent studies conducted under different experimental conditions; direct quantitative comparison across studies is, therefore, not strictly applicable. N/A indicates that the corresponding data were not reported in the original reference.
